# Single-cell analysis of salt-induced hypertensive mouse aortae reveals cellular heterogeneity and state changes

**DOI:** 10.1038/s12276-021-00704-w

**Published:** 2021-12-03

**Authors:** Ka Zhang, Hao Kan, Aiqin Mao, Li Geng, Xin Ma

**Affiliations:** Wuxi School of Medicine, Jiangnan University, Wuxi, 214000 China

**Keywords:** Hypertension, Aortic diseases

## Abstract

Elevated blood pressure caused by excessive salt intake is common and associated with cardiovascular diseases in most countries. However, the composition and responses of vascular cells in the progression of hypertension have not been systematically described. We performed single-cell RNA sequencing on the aortic arch from C57BL/6J mice fed a chow/high-salt diet. We identified 19 distinct cell populations representing 12 lineages, including smooth muscle cells (SMCs), fibroblasts, endothelial cells (ECs), B cells, and T cells. During the progression of hypertension, the proportion of three SMC subpopulations, two EC subpopulations, and T cells increased. In two EC clusters, the expression of reactive oxygen species-related enzymes, collagen and contractility genes was upregulated. Gene set enrichment analysis showed that three SMC subsets underwent endothelial-to-mesenchymal transition. We also constructed intercellular networks and found more frequent cell communication among aortic cells in hypertension and that some signaling pathways were activated during hypertension. Finally, joint public genome-wide association study data and our single-cell RNA-sequencing data showed the expression of hypertension susceptibility genes in ECs, SMCs, and fibroblasts and revealed 21 genes involved in the initiation and development of high-salt-induced hypertension. In conclusion, our data illustrate the transcriptional landscape of vascular cells in the aorta associated with hypertension and reveal dramatic changes in cell composition and intercellular communication during the progression of hypertension.

## High blood pressure: how salt affects cells in the aorta

Salt-induced high blood pressure involves dramatic changes in cell composition and communication in blood vessels, a genetic analysis reveals. Over 99% of the world’s adult population has an excessive salt intake, which is associated with hypertension and numerous cardiovascular diseases. Xin Ma and co-workers at Jiangnan University in Wuxi, China, performed single-cell RNA sequencing on aortas from mice that were fed a high-salt diet, and combined their results with genome-wide association studies. They revealed that as hypertension progresses the proportion of smooth muscle cells, cells lining blood vessels, and T cells increases, while several signaling pathways are enriched and new pathways become activated. They also identified 21 associated genes, including *Nox4*, which promotes collagen synthesis and leads to arterial stiffness. This new understanding provides potential targets for prevention and treatment of hypertension.

## Introduction

For 99.2% of the global adult population, individual salt (NaCl) intake exceeds the maximum limit recommended by the World Health Organization^1,2^. A high dietary intake of salt is associated with poor health outcomes, including cardiovascular disease^3^, kidney disease^4^, autoimmunity^5^, stomach cancer^6^, and dementia^7^. Hypertension is one of the important factors contributing to these diseases^3,8^. Although it is well established that high-salt-induced hypertension can lead to endothelial dysfunction^9^, vascular inflammation^10^, reduced arterial vasodilator capacity^11^, increased arterial stiffness^12^, and the promotion of vascular remodeling^13^, a detailed description of the heterogeneity and the relative contributions of different vascular cells in normal aortas and those in high-salt-induced hypertension is lacking.

Recently, the development of single-cell RNA sequencing (scRNA-seq) has made it possible to systematically study heterogeneity across a large number of individual cells in normal and diseased tissue^14,15^. Previously, the cell composition and heterogeneity of murine and human aortae in different diseases have been described using unbiased scRNA-seq^16–19^. These studies characterized the extensive transcriptional and functional heterogeneity of vascular cells during the progression of diseases.

Elucidating the alterations in cell composition, heterogeneous identities, and changes in cell function in normal aortas and after induction of high-salt-induced hypertension is helpful for understanding the progression of hypertension and provides potential targets for prevention and treatment. In the present study, we performed scRNA-seq on normal and high-salt-induced hypertension aortas and characterized the cellular heterogeneity, changes in transcriptomic profiles, and functional states of vascular cells. Our analyses also revealed potentially important cell subpopulations and intercellular communication in the progression of hypertension. In addition, we integrated our scRNA-seq data with public genome-wide association study (GWAS) data to identify cell type-specific expression of GWAS risk loci for hypertension and reveal genes that are involved in hypertension initiation and development.

## Materials and methods

### Experimental animals

Animal studies were approved by the Animal Care Committee of Jiangnan University (approval number: JN. No20190430c1801231[86]) and complied with the National Institutes of Health (NIH) guide for the care and use of laboratory animals. Mice were housed in stock-holding rooms under specific pathogen-free conditions. Male C57BL6/J mice (5 weeks old) were fed a chow diet (0.3% NaCl) or a high-salt diet (8% NaCl) to induce hypertension^20^. Mice had free access to water and food throughout the study. Systolic and diastolic blood pressure were measured using a noninvasive tail-cuff system (NIPB-2 blood pressure monitor, Columbus Instruments, Columbus, OH)^20^. Blood pressure and body weight were measured at least weekly until stable.

### Preparation of single-cell suspension

Mice were euthanized through CO_2_ inhalation, and aortic arches were rapidly removed and transferred to ice-cold phosphate-buffered saline (PBS). After removing the perivascular adipose tissue, the arches were finely cut into ~1 mm pieces and incubated in 1× aortic dissociation enzyme solution containing 0.2% type I collagenase (Worthington Biochemical Corp., Lakewood, NJ) and 200 U/ml DNase (Sigma, USA) for 20 min at 37 °C. The reaction was deactivated by adding 10% FBS. The suspension was then passed through a 40 μm filter (STEMCELL Technologies China Co., Ltd, Shanghai), treated with ACK lysis buffer for 5 min at room temperature, and washed once with PBS.

### Single-cell RNA sequencing

Single-cell suspensions were processed through the Chromium platform (10x Genomics, Pleasanton, CA, USA), and 3′ gene expression v3 libraries were constructed with Chromium^TM^ Single Cell 3′ Reagent Kits (10x Genomics) and sequenced on an Illumina HiSeq X Ten platform. Briefly, single-cell suspensions mixed with reverse transcription reagents along with gel beads and oil were loaded onto 10X Chromium Chip B to generate single-cell gel beads in emulsions (GEMs). To capture 5000 cells per library, ~8000 cells were added to each channel. GEM-RT–PCR was performed in an S1000^TM^ thermal cycler (Thermo Fisher Scientific) to barcode cDNA using the following program: 53 °C for 45 min, 85 °C for 5 min; held at 4 °C. Next, cDNA was amplified by PCR. Subsequently, the amplified cDNA was fragmented, end-repaired, A-tailed, and ligated to an index adaptor, and then the library was amplified. Final libraries were sequenced on an Illumina HiSeq X Ten.

### Preprocessing of sequencing data

Raw single-cell RNA-seq data were demultiplexed and processed using CellRanger v.3.0.2 (10x Genomics). The software function mkfastq was used to generate fastq files, which were then aligned to the reference mouse mm10 transcriptome (GRCm38), filtered, and counted using the CellRanger function count to generate the gene-barcode matrix. Expression matrices were loaded into R software for further data analysis.

### scRNA-seq data processing and clustering

The R package Seurat (version 3.1.2) was used for subsequent analysis^21^. The following quality control criteria were used to exclude poor-quality cells: gene count per cell >600 and <4000, percentage of mitochondrial genes <10%, percentage of ribosomal genes <40%, and no *hemoglobin subunit beta* gene detected in the cell. The remaining data were used for follow-up analysis. The following steps were performed according to the Seurat manual: data were log normalized and scaled, the most variable genes (2000 genes) for all samples were used for linear dimensional reduction (principal component analysis, PCA), the “FindCluster” function of Seurat (resolution = 0.4) was used for cluster analysis, and nonlinear dimensional reduction (T-distributed stochastic neighbor embedding; tSNE) was used to visualize the results in tSNE plots. Conserved (marker) genes in clusters were identified using the ‘FindAllMarkers’ function of Seurat with default parameters. Cell types were identified.

### Differential gene analysis and functional annotation

Differential gene expression between the control and high-salt groups was analyzed by using the “FindMarkers” function of Seurat with the Wilcox test. Genes with adjusted *p* values < 0.05 and |logFC| > 0.25 were considered to be differentially expressed genes (DEGs). Gene set enrichment analysis of the DEGs was performed using the Bioconductor R package fgsea (v1.8.0).

### Module score and cell cycle analysis

For module score analysis, gene sets were curated from the MSigDB database and the literature. Gene sets for each module are listed in Supplementary Table 1. For some gene sets, we used Ensembl BioMart (http://www.ensembl.org/biomart/martview/) to convert human genes to their orthologous mouse counterparts. The function “AddModuleScore” from Seurat was used to calculate the module score of each cell. For cell cycle phase scoring, the CellCycleScoring function of Seurat was used.

### Analysis of cell–cell communication

Cell–cell communication was assessed using CellChat (Version 0.5.0) with default settings^22^. Briefly, CellChat input files were extracted from the Seurat V3 object, and the “computeCommunProb” function from CellChat was used to compute the communication probability and infer the cellular communication networks of the control and hypertension groups. The CellChat functions “netVisual_aggregate”, “plotGeneExpression”, and “netAnalysis_dot” were used to visualize the signaling pathways with hierarchy plots, signaling gene expression with violin plots, and incoming communication patterns of target cells with dot plots.

### Immunofluorescence

Slides of aortic tissue were fixed with 4% paraformaldehyde for 30 min at room temperature and permeabilized in 0.1% PBS-Triton for 30 min. The samples were blocked in 5% BSA/PBS/0.1% Triton-X 100 for 1 h and then incubated with the primary antibodies anti-Kruppel-like factor 4 (Klf4, ab214666), anti-neural cell adhesion molecule (Ncam1, ab28486), anti-Ki67 (ab15580), anti-alpha-smooth muscle actin (ab7817) (all from Abcam, USA), and anti-vitronectin (Vtn, MA5-24083, Invitrogen, USA). After several PBS washes, the slides were incubated for 2 h at room temperature with the secondary antibodies AF647 donkey anti-mouse and AF568 donkey anti-rabbit (Invitrogen, USA). Immunofluorescence was assessed using a Zeiss LSM 880 confocal laser-scanning microscope (Carl Zeiss Microscopy, Jena, Germany). The fluorescence values were measured with ImageJ software (developed at the National Institutes of Health). At least 100 cells were analyzed for each group.

### ROS measurement in en face endothelium of mouse aortas

After isolation, aortic segments were incubated with DHE (Beyotime Biotechnology, China) at 5 μmol/l for 30 min in extracellular medium consisting of 121 mM NaCl, 5 mM NaHCO_3_, 10 mM Na-HEPES, 4.7 mM KCl, 1.2 mM KH_2_PO_4_, 1.2 mM MgSO_4_, 2 mM CaCl_2_, and 10 mM glucose, pH = 7.4. After PBS washing, the aorta rings were cut open, and the endothelium was placed between two coverslips for imaging by a confocal microscope as described earlier^23^.

### Western blotting

Protein samples prepared from mouse aortic arch homogenates were electrophoresed and separated on 15% SDS-polyacrylamide gels and transferred to a PVDF membrane (Millipore), which was subsequently blocked with 1% BSA in 0.05% Tween-20 TBS and incubated overnight at 4 °C with the following primary antibodies: anti-TWEAK (1:500, SAB, China) and anti-TWEAKR (1:500, SAB, China). Anti-GAPDH (1:5000, Invitrogen, USA). The membrane was washed and incubated with the appropriate secondary antibodies for 2 h at room temperature; anti-mouse antibodies were from Invitrogen (USA). Protein bands were detected by FluorChem E (Protein Simple, USA).

### Statistical analysis

Data were analyzed with Prism 8 (GraphPad Software, Inc.) and are represented as mean ± standard error of the mean. Unpaired Student’s *t* test was performed to determine the *p* values, and a *p* value < 0.05 was considered statistically significant. Gene set scores were compared between cell populations of samples using the Mann–Whitney *U* test.

## Results

### Profiles of single aortic cells in control and high-salt-induced hypertensive mice

**Fig. 1:**
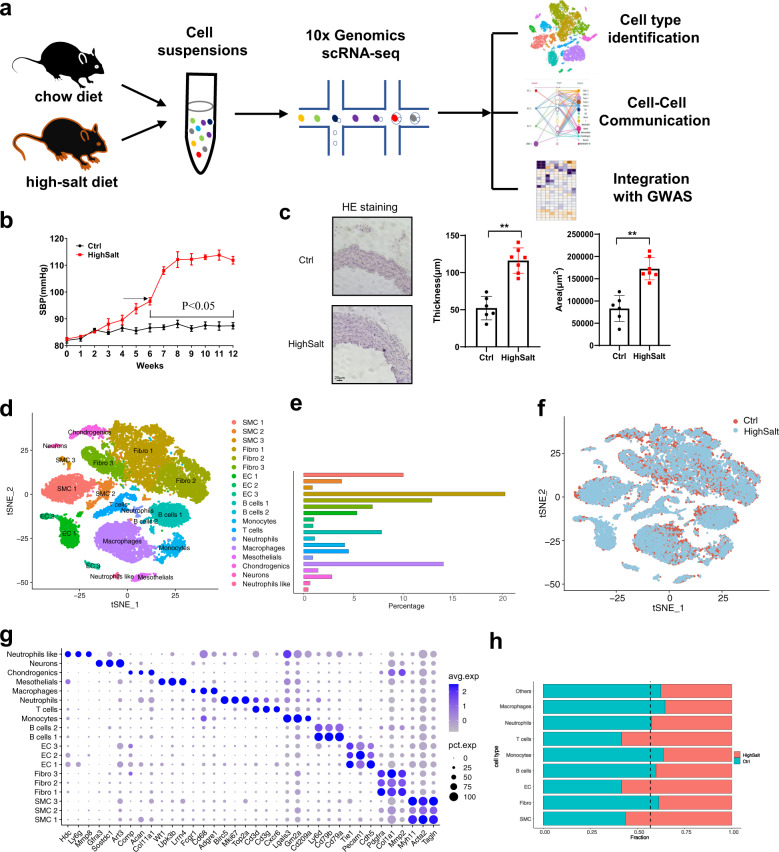

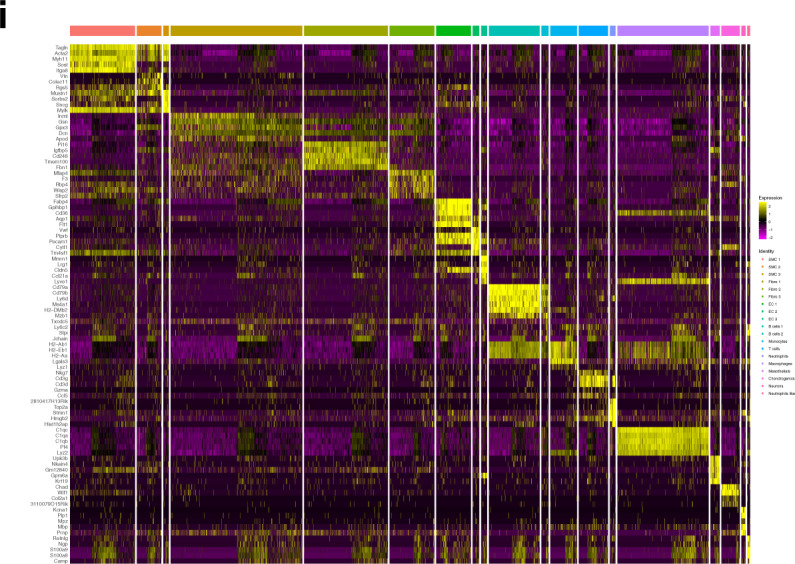
Cell types in mouse aortic arch cells delineated by single-cell RNA sequencing (scRNA-seq). **a** Overview of the experimental approach. **b** Blood pressure over 12 weeks in mice fed a high-salt diet. **c** A high-salt diet induces increased aortic wall thickness. Representative H&E staining images are shown in the left panel, and quantitative data are shown in the middle and right panels. Scale bar: 20 μm. Data are represented as mean ± SEM. ^**^p < 0.01 by Student’s *t* test. *n* = 6–8. **d** T-distributed stochastic neighbor embedding (t-SNE) plot of aggregate aortic arch cells from chow diet (*n* = 4) and high-salt diet (*n* = 3) mice. Colors denote 19 distinct cell types. **e** Proportions of cell types in the aortic arch. **f** t-SNE plot of aggregate aortic arch cells with colors denoting different groups. Control, *n* = 13,017 cells. Hypertension, *n* = 9900 cells. **g** Dot plot depicting 3 marker genes in aggregate aortic arch cell clusters. **h** Heatmap of the top 5 genes (by average log(fold change)) per cluster. **i** Bar graph of the composition of each cell type (dashed black line, expected proportion of cells in the control group (total number of cells in the control group divided by total number of cells in all samples)).

We established a high-salt-induced hypertension model in C57BL/6J mice, as previously reported^20^. The high-salt diet markedly increased blood pressure and promoted vascular remodeling after 10 weeks, as evidenced by increased thickness and area of aorta (Fig. 1b, c). Therefore, after 10 weeks of the high-salt diet, single-cell transcriptomic profiles from the aortic arch of hypertensive and control mice were generated.

We sequenced a total of 36,076 cells from aortic arch cell suspensions from 3 hypertensive and 4 control mice (Fig. 1a). Using stringent quality control, a total of 22,917 qualified cells were obtained for further analysis. Data integration and unbiased clustering of these cells identified 19 clusters, which were then visualized using the tSNE algorithm (Fig. 1d–f). We generated cluster-specific marker genes to define the identity of each cluster, including smooth muscle cells (SMCs) (Myh11; Acta2; Tagln), fibroblasts (Pdgfa; Col1a1; Mmp2), endothelial cells (ECs) (Tie1; Pecam1; Cdh5), B cells (Ly6d; Cd79a; Cd79b), monocytes (Cd209a; Gm2a; Lgals3), T cells (Cd3g; Cd3d; Cxcr6), neutrophils (Birc5; Mki67; Top2a), macrophages (Cd68; Adgre1; Fcgr1), mesothelial cells (Wt1; Upk3b; Lrrn4), chondrogenic cells (Acan; Col11a1), Schwann cells (Gfra3; Art3; Sostdc1), and a small population of cells with a neutrophil-like gene expression profile (such as Hdc and Ly6g), but did not express the hallmarks of neutrophils (Fig. 1g and Supplementary Fig. 1). In total, we identified one cluster each of macrophages, mesothelial cells, chondrogenic cells, Schwann cells, T cells, monocytes, and neutrophil-like cells; two of B cells; and three of SMCs, ECs, and fibroblasts (Supplementary Fig. 2). Quantitatively, SMCs, fibroblasts, and macrophages were the largest components (Fig. 1e). The top 5 marker genes (sorted by *p* value) for each cluster were identified, and these genes were visualized using a heatmap (Fig. 1i). Next, we examined the source composition of each cell type (Fig. 1h) and found that hypertensive tissue contributed more cells than expected to T cells, SMCs, and ECs. Furthermore, we determined that a high-salt-diet-induced expansion of the total T cell, SMC, and EC populations compared to the chow diet group (Supplementary Fig. 3). The increase in these cells may involve vascular remodeling and vascular inflammation, which is consistent with previous reports^24–26^.

### Changes in the composition and function of endothelial cell populations in the hypertensive aortic wall

**Fig. 2:**
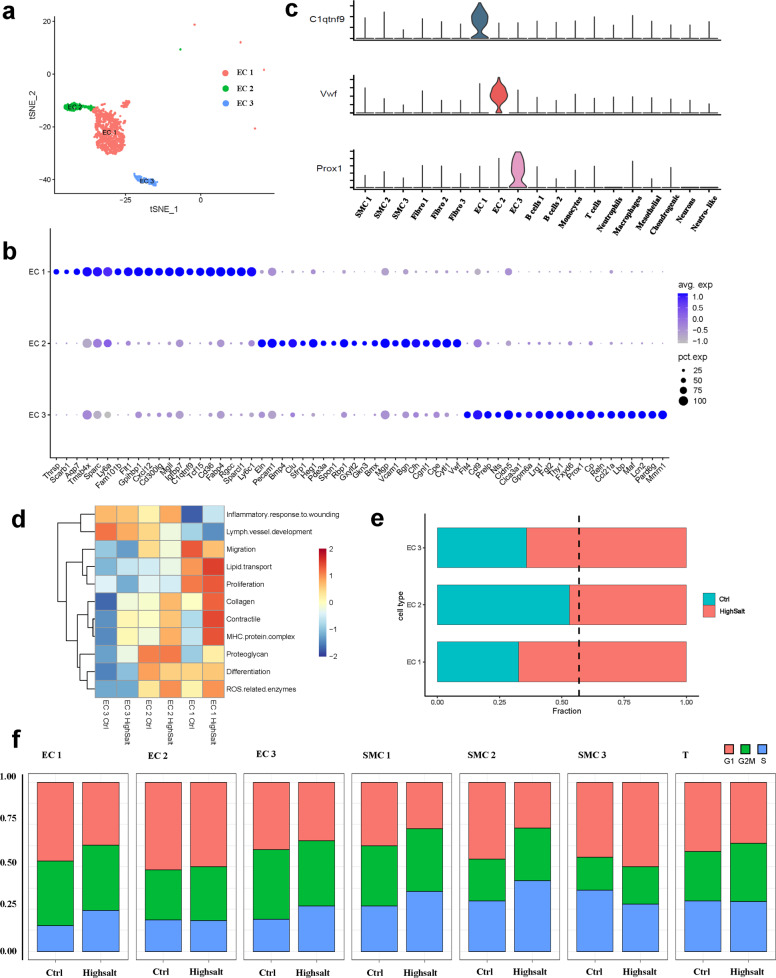
Characteristics of aortic ECs and their changes in hypertension. **a** t-SNE plot of the EC subpopulations from control and hypertensive tissue (control, *n* = 624 ECs; hypertension, *n* = 1056 ECs. EC 1, *n* = 1107 cells; EC 2, *n* = 302 cells; EC 3, *n* = 271 cells). **b** Expression levels of marker genes associated with 3 EC subpopulations across the 19 clusters. **c** Dot plot of the top 20 genes with specific expression for each EC subpopulation. **d** Module scores of 11 features (or functions) in control and hypertensive aortic EC subpopulations. **e** Bar graph of the composition of each EC subpopulation (dashed black line, expected proportion of cells in the control group). **f** Cell cycle stage distribution of 7 subpopulations from control and hypertensive tissue.

Cluster analysis of all cells from both control and hypertensive aortas identified three distinct clusters of ECs displaying classical endothelial markers Cdh5, Pecam1, and Tie1 (Fig. 2a), and we characterized these subpopulations by identifying the marker genes that differentiate them (Fig. 2b). Each of the three EC clusters expressed distinct and nonoverlapping markers, suggesting the presence of discrete subpopulations. The results of our cluster analysis and the gene expression of each cluster were also consistent with the previous report of Kalluri et al^27^. We identified the genes encoding C1q And TNF Related 9 (C1qtnf9), von Willebrand factor (vWF), and Prospero homeobox 1 (Prox1) as the key marker genes for clusters EC 1, EC 2, and EC 3 (Fig. 2c).

To obtain a more comprehensive understanding of the functional heterogeneity of each EC cluster and the effect of hypertension on the functions, we evaluated several features (or functions). The EC 3 cluster strongly expressed the lymphatic endothelium marker gene Prox1 and showed higher module scores of lymph vessel development than the EC 1 and EC 2 clusters (Fig. 2b, d)^28^. It has been suggested that EC 3 includes lymphatic ECs. Between the 2 clusters of blood ECs, the EC 1 cluster showed greater migration and proliferation (Rgcc and Flt1)^29–31^, lipid transport, and lipid metabolism (Cd36, Fabp4, Thrsp, and Gpihbp1) than the EC 2 cluster (Fig. 2b, d and Supplementary Fig. 4d, i, k)^32–34^. The EC 2 cluster strongly expressed proteoglycan genes (Clu and Bgn). Furthermore, the EC 2 cluster showed higher differentiation and inflammatory response to wounding than the EC 1 cluster (Fig. 2b, d and Supplementary Fig. 4c, e, f). Concerning the changes in features (or functions) of EC 1 and EC 2 under hypertension, we found that the clusters shared a common response, in which the expression of reactive oxygen species (ROS)-related enzymes, collagen, contractility, and histocompatibility complex genes were all upregulated (Fig. 2d and Supplementary Fig. 4a, b, g, h, j). Indeed, ROS production measured by DHE fluorescence in the en face endothelium of the aorta was higher in the high-salt diet mice (Supplementary Fig. 5a, b).

During the progression of hypertension, the proliferation of blood ECs was mainly due to expansion of the EC 1 cluster, while the proportion of the EC 2 cluster did not change (Fig. 2e). Cell cycle analysis indicated that EC 1 and EC 3 cells had a greater population in the S stage in hypertensive aortas than in controls, while the EC 2 cluster did not significantly differ (Fig. 2f), indicating that the EC 1 and EC 3 cluster have a higher proliferation rate in hypertensive aortas.

### Changes in the composition and function of SMC populations in hypertensive aortic walls

**Fig. 3:**
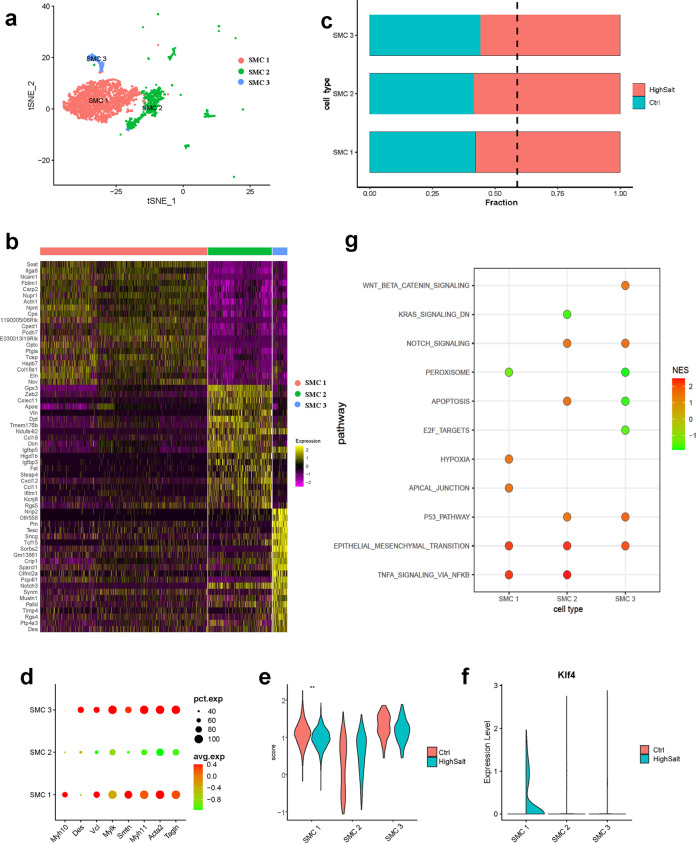
Characteristics of aortic SMCs and their changes in hypertension. **a** t-SNE plots of the SMC subpopulations from control and hypertensive tissue (control, *n* = 1422 SMCs. hypertension, *n* = 1949 SMCs. SMC 1, *n* = 2296 cells; SMC 2, *n* = 874 cells; SMC 3, *n* = 202 cells). **b** Heatmap showing the top 10 genes (by average log(fold change)) for each SMC cell subpopulation. **c** Bar graph of the composition of each SMC subpopulation (dashed black line, expected proportion of cells in the control group). **d** Dot plots of typical SMC markers for 3 SMC subpopulations from the control. **e** Expression of contractile SMC markers for 3 SMC subpopulations from control and hypertensive tissue. ^***^Mann–Whitney *U* test *p* < 0.001 vs. SMC 1, HighSalt. **f** Violin plots of the Klf4 gene associated with phenotypic modulation of SMCs. **g** The results of gene set enrichment analysis (GSEA) using a hallmark gene set showing enriched differentially expressed gene (DEG) counts between the control and hypertension groups.

We identified 3 types of SMCs (clusters SMC 1, SMC 2, and SMC 3) that expressed the SMC marker genes Myh11, Acta2, and Tagln (Figs. 1g and 3a), while their gene expression profiles were not similar. Regulatory cell adhesion-related genes such as neural cell adhesion molecule 1, nephronectin (Npnt), and protocadherin 7 were selectively expressed by SMC 1. Cells in the SMC 1 cluster also strongly expressed cysteine-rich protein 2, which inhibits vascular SMC migration^35^. In SMC 2, insulin-like growth factor binding protein-5 (Igfbp5), Igfbp3, and interferon-induced transmembrane protein 1 (Ifitm1) were strongly expressed and have been associated with SMC proliferation^36,37^. SMC 2 cells also expressed chemokine ligand-encoding genes Cxcl12, Ccl11, and Ccl19, suggesting that this type of SMC may be involved in inflammatory responses. The smallest cluster, SMC 3, exhibited the strongest expression of nuclear receptor interacting protein 2, phospholamban, and tescalcin. The SMC 1 and SMC 2 clusters more strongly expressed ECM-related genes (Eln, Col18a1, and Dcn) than the SMC 3 cluster (Fig. 3b).

Compared with control tissue, SMC 1, SMC 2, and SMC 3 increased during hypertension development (Fig. 3c). Of note, the increase in the SMC 1 and SMC 2 clusters was consistent with our cell cycle analysis and Ki67 immunofluorescence staining results (Fig. 2f and Supplementary Fig. 6a–d). However, in the SMC 3 cluster, fewer cells were assigned to the S stage in hypertensive tissue than in controls (Fig. 2f), suggesting that cell proliferation is not the main reason for the increase in the proportion of SMC 3 in hypertensive tissue.

Then, we examined the expression of typical SMC markers in each cluster (Supplementary Table 2). Both the SMC 1 and SMC 3 clusters strongly expressed contractility-related genes such as Myh11, Acta2, and Tagln, and in the SMC 2 cluster, they were lower (Fig. 3d). Using the previously identified gene signatures for the contractile phenotype SMCs to generate a score for SMC clusters in both the hypertension and control groups, we found that the expression of contractile phenotype-related genes was lower in SMC 1 under hypertension, whereas there was no significant difference between the hypertension and control groups in SMC 2 and SMC 3 (Fig. 3e). Combined with the expression profile analysis, our results suggested that SMC 1 is a contractile subpopulation, SMC 2 is a synthetic subpopulation, and SMC 3 needs further study. In addition, Klf4 was detected in the SMC 1 cluster of the hypertension group^38^, indicating phenotypic modulation of SMC 1 (Fig. 3f), and the upregulation was confirmed by immunofluorescence staining (Supplementary Fig. 7a, b).

To further analyze the key changes in SMCs under hypertension, gene set enrichment analysis was performed on the DEGs between the hypertension and control groups using the hallmark gene sets from MsigDB as background genes. We found that endothelial–mesenchymal transition was higher in SMC clusters from hypertensive tissue (Fig. 3g). To validate these findings, the expression of the SMC marker Acta2 was evaluated in histological sections and showed cellular expression in the intima of hypertensive aortae (Supplementary Fig. 8a, b), indicating the presence of endothelial-to-mesenchymal transition in hypertensive aortic cells^39^. Therefore, we hypothesized that part of the increase in the proportion of SMCs in hypertensive tissues may be due to the transition of ECs.

### Changes in the composition and function of T cell populations in hypertensive aortic walls

To identify changes in T cells from the hypertensive aortic arch, we performed integrative unsupervised reclustering of the T cell population from all aortic arch tissue samples (Supplementary Fig. 9a). We identified 4 types of CD8^+^ T cells (Cd8a), Th17 (Rorc), Th2 (Gata3), and natural killer (NK) T cells (Klrb1c) (Supplementary Fig. 9b,c).

Compared with control tissue, Th17 and Th2 cells increased during hypertension development, while the proportions of NK T and CD8^+^ T cells did not change (Supplementary Fig. 9d). To further understand the functional changes of T cells, we determined the mean expression of cytokine and receptor genes in T cell subclusters in the control and hypertensive aortic arches and found that cytokine gene expression was higher in the hypertensive aortic arch than in the control, suggesting that cytokines may play an important role in the development of hypertension (Supplementary Fig. 9e). Cell cycle analysis indicated that the parameters in T cells did not significantly change in hypertensive and control tissue. We calculated the expression of all chemokine genes according to T cell subclusters and identified Th17 and Th2 clusters with significantly increased chemokine gene expression in hypertensive tissues (Supplementary Fig. 9f). This suggested that the increased proportion of T cells in hypertensive tissue may be attributed to the attraction of distal T cells.

### Inference and analysis of cell–cell communication

**Fig. 4:**
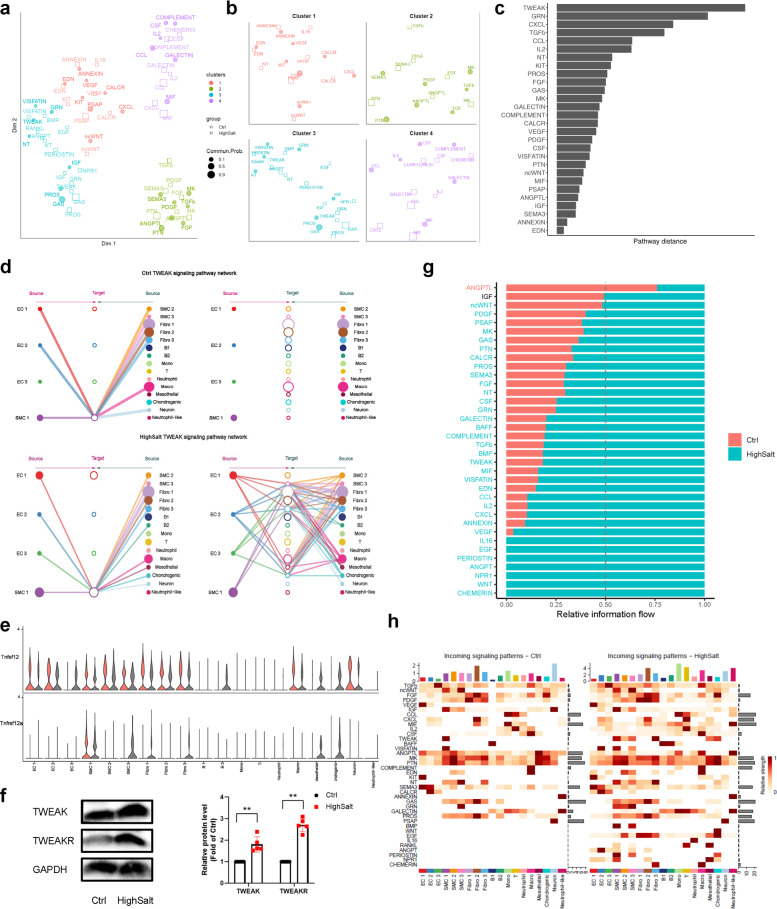
Comparison analysis of cell–cell communications between control and hypertensive aortas. **a** Jointly projecting signaling pathways from control and hypertensive tissue into a shared two-dimensional manifold according to their functional similarity. Each dot indicates the communication network of one signaling pathway. Dot size is proportional to the communication probability. Different colors represent different groups of signaling pathways. **b** Enlarged view of each pathway group. **c** Ranked overlapping signaling pathways between control and hypertensive tissue; a greater distance implies a larger difference in the communication network between control and hypertension. **d** Hierarchical plot showing the inferred intercellular communication network of the TWEAK signaling pathway in the control and hypertension groups. **e** Violin plot showing the expression distribution of signaling genes involved in the inferred TWEAK signaling network. **f** Western blot of TWEAK signal-related protein expression in aortic arch tissue. Data are represented as mean ± SEM. ^**^p < 0.01 by Student’s *t* test. *n* = 6–8. **g** Significant signaling pathways ranked based on differences in the overall information flow within the inferred networks between the control and hypertension groups. Red, top pathways enriched in control aortas; black, equally enriched in control and hypertension; green, enriched in hypertension. **h** Comparison of incoming signaling patterns of cells between the control and hypertension groups. The color is proportional to the contribution score computed from pattern recognition analysis. A higher score implies that the signaling pathway is more enriched in the corresponding cell group.

We next compared the cell–cell communication patterns between hypertensive and control tissue based on CellChat (Version 0.5.0)^22^. First, the intercellular communications in hypertension and control datasets were calculated separately, and then the signaling pathways from both datasets were clustered into groups based on their functional similarity. We identified four pathway groups (Fig. 4a, b). Group #1, which included the ncWNT, EDN, CALCR, and KIT pathways, largely represented signaling from fibroblasts and ECs. Groups #2 and #3 were dominated by growth factors, such as TGFβ, FGF, PDGF, ANGPTL, EGF, IGF, and TWEAK. Group #4 was dominated by inflammatory pathways, such as IL2, CCL, CXCL, and CSF.

To determine whether the signaling between two datasets was similar, we computed the Euclidean distance between any pair of shared signaling pathways in the shared two-dimensional manifold and found a large distance for TWEAK pathways (Fig. 4c). We specifically examined how TWEAK communications change in the development of hypertension (Fig. 4d, e). Compared to control tissue, only SMC 1 cells were TWEAK targets; in hypertensive tissue, SMC 2, SMC 3, Fibro 3, mesothelial cells, and chondrogenic cells gained TWEAK responsiveness. Furthermore, EC 3, mesothelial cells, and chondrogenic cells emerged as new minor sources of TWEAK signaling, helping to drive an overall increase in the complexity of the TWEAK communication network. As expected from scRNA-seq, upregulation of TWEAK and TWEAKR protein expression was observed in hypertensive aortic tissue (Fig. 4f). This suggested that TWEAK pathways undergo changes in hypertension, consistent with their reported role in pathological vascular remodeling^40^.

We also compared the information flow for each signaling pathway between hypertensive and control tissue (Fig. 4g). Unexpectedly, 26 out of 29 pathways were more active in hypertensive tissue than controls. In addition, 9 pathways were specifically active in the hypertension group, including signaling pathways that regulate vascular development in the embryo and were reactivated following vascular injury (EGF, BMP, RANKL, WNT, and ANGPT)^41–44^ and pathways that have not been fully studied in the cardiovascular system, such as PERIOSTIN, NPR1, and CHEMERIN.

Moreover, we examined the detailed changes in the signal receptor levels of all significant pathways (Fig. 4h). The results showed that the overall expression of signaling pathway receptors in hypertensive tissue was significantly increased. The changes in the signal pathway targets of the EC1 and EC3 clusters were similar: they maintained incoming signaling patterns for targets such as TGFβ, ncWNT, and VEGF, signaling was turned on for IL2, EDN, CALCR, PROS, NPR1, ANGPT, and EGF, and the ANGPTL pathway was decreased. Some signaling pathways changed their receptors in EC 2 cells: they were (i) decreased (ANGPTL and SEMA3), (ii) increased (TGFβ, ncWNT, CXCL, and MIF), or (iii) turned on (IGF, IL2, CSF, VISFATIN, and ANGPT). On the other hand, the incoming signaling of SMC clusters was prominently altered. Some signaling pathways were reactivated, such as the NT, GRN, BMP, PERIOSTIN, and NPR1 pathways in the SMC 1 cluster, the TWEAK, GRN, and EGF pathways in the SMC 2 cluster, and the TWEAK, VISFATIN, BMP, and PERIOSTIN pathways in the SMC 3 cluster. In addition, the expression of CXCL signaling pathway receptors was increased in T cells in hypertensive tissue, consistent with the results in Supplementary Fig. 9e, f.

### Association of the DEGs in hypertensive tissue with the GWAS results

**Fig. 5:**
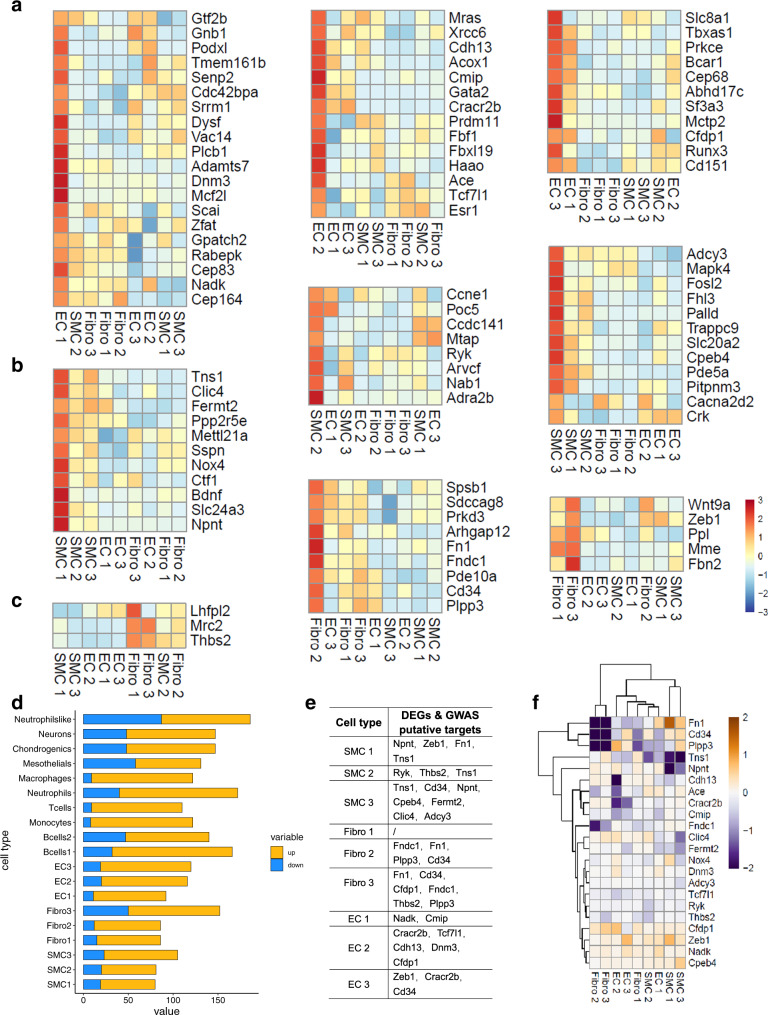
Association between risk genes for high blood pressure and aortic cells. **a**–**c** Blood pressure GWAS-related genes enriched in ECs (**a**), SMCs (**b**), and fibroblasts (**c**). Mean expression values of the genes calculated in each cluster. Each row is a gene, and each column represents a single cell type. **d** Differentially expressed gene (DEG) counts between the hypertension and control groups in each cluster (blue, downregulated genes; red, upregulated genes). **e** DEGs identified as blood pressure GWAS-related genes according to cell cluster. **f** Heatmap showing differences in the average expression of candidate genes, (**e**) between hypertension and control groups according to cell cluster. A higher row-scaled mean expression score indicates increased gene expression in hypertension.

Population-based genome-wide association studies (GWASs) have revealed 107 independent loci associated with blood pressure traits^45^. We used single-cell transcriptomic data to relate the genetic risk of high blood pressure with cell type-specific expression in aortic tissue. We found that 93 homologous mouse genes were expressed in aortic tissue. Of the 93 genes, 45 showed the strongest expression in ECs (Fig. 5a), distributed in the three clusters (20 in EC 1, 14 in EC 2, and 11 in EC 3). Thirty-one genes were preferentially expressed in SMCs (11 in SMC 1, 8 in SMC 2, and 12 in SMC 3) (Fig. 5b), such as Nox4, Ctf1, Arvcf, and Fosl2. Only 17 blood pressure-related genes were strongly expressed in fibroblasts (3 in Fibro 1, 9 in Fibro 2, and 5 in Fibro 3) (Fig. 5c). Our results suggested that ECs and SMCs make major contributions to genetic associations with high blood pressure.

To further understand the pathogenesis of high-salt-induced hypertension and develop potential treatments, we sought to identify the DEGs related to genes associated with blood pressure traits. We compared data between hypertensive and control tissue and found that more of the DEGs were upregulated (Fig. 5d). Then, we overlapped the DEGs with candidate genes related to blood pressure traits. From these candidate genes, we identified 21 genes (such as fibronectin 1 (Fn1), Cd34, Npnt, Tns1, Nox4, and Ace) that were differentially expressed in ECs, SMCs, and fibroblasts between hypertensive and control tissue (Fig. 5e). We further studied the detailed changes in the 21 genes (Fig. 5f). The results showed that in hypertensive tissue, Plpp3, Tns1, Npnt, Cdh13, Cracr2b, Cmip, Fndc1, Clic4, Fermt2, Adcy3, Tcf7l1, Ryk, and Thbs2 were decreased and Nox4, Dnm3, Cfdp1, Zeb1, Nadk, and Cpeb4 were increased. However, Fn1 and Cd34 expression was decreased in the Fibro 2 and Fibro 3 clusters but increased in the SMC 1 cluster. The effects of Fn1 and Cd34 on hypertension warrant further study.

## Discussion

scRNA-seq technology has undergone rapid development in recent years, providing a powerful approach to dissect cellular heterogeneity in cardiovascular disease. Various studies have described the heterogeneity of vascular cells of the aorta in health and diseases, including atherosclerosis^16^, aortic aneurysm^18^, spontaneous hypertension^19^, and metabolic pathologies. However, the aortic cellular composition and heterogeneity associated with high-salt-induced hypertension remain largely unknown. In this study, we used scRNA-seq methods to reveal the transcriptional profiles in 16,972 individual cells from the aortas of healthy and hypertensive mice.

Our data revealed several fundamental discoveries. We described changes in the composition and functional state of cell subpopulations in the aorta of hypertensive mice in a more comprehensive way than previously reported. Previous studies on vascular cell changes in hypertension mainly focused on total SMCs, ECs, or fibroblasts to study the mechanism of cell proliferation and dysfunction. Using scRNA-seq, we confirmed the relative contributions of three subpopulations of SMCs and ECs to vascular remodeling. We systematically revealed the signaling pathway changes of intercellular communication in hypertension. It is well known that many signaling pathways are activated during this disease. However, the changes in signaling pathways in hypertensive arteries are not completely clear. We showed that 26 signaling pathways were more enriched and 9 signaling pathways were activated in hypertensive arteries. Furthermore, we mapped genes based on GWAS risk loci for blood pressure traits and identified target genes that are involved in hypertension initiation and development. These results show a changing, diverse cellular landscape in hypertensive arteries.

During the progression of hypertension, one dramatic change that occurs in arteries is vascular remodeling. This process is regulated by many factors, including increased SMCs and EC proliferation and migration^25,26^, endothelial dysfunction^9^, increased inflammatory cell recruitment^46^, and excess ROS production^47^. Based on our scRNA-seq data, we detected subpopulations of ECs and SMCs similar to those previously reported. The proportions of EC 1, EC 3, and 3 SMC clusters increased in hypertensive arteries due to proliferation or endothelial–mesenchymal transition. In addition, compared with control tissues, ROS and extracellular matrix production increased the EC 1 and EC 2 subsets, and contractile SMCs (SMC 1) developed a synthetic phenotype in the hypertensive aorta. This suggests that the mechanisms of vascular remodeling induced by cell subsets are different.

In addition to the changes in EC and SMC clusters, our study also describes intercellular communication in the hypertensive aorta and provides new insights into the progression of hypertension. We have shown more frequent cell communication among aortic cells in hypertension; this indicates that many of the pathological features of hypertension are caused by excessive activation of signaling pathways. Several pathways have been reported to be associated with vascular diseases: the TWEAK, TGFβ, BMP, and PERIOSTIN pathways participate in vascular remodeling^40,43,48^, and the IL2, CCL, CXCL, IL16, and VISFATIN pathways are involved in vascular inflammation. These examples provide support for inferred cellular signaling pathways that we found and that have not previously been associated with hypertensive vascular diseases. It is expected that the cellular communication information provided in this study will provide specific pathways to target for the development of new treatments for hypertension.

Integration of single-cell genomics and the GWAS results is helpful for understanding the pathogenesis and potential therapeutic targets of complex diseases, including hypertension. Our analysis showed preferential expression of blood pressure-associated genes in ECs, SMCs, and fibroblasts. We also identified 21 DEGs (e.g., Fn1, Cd34, Plpp3, Tns1, Nox4, and Npnt) that may be involved in the development of hypertension. Specifically, our results showed that the expression of Nox4 increased more strongly in SMCs than in ECs. This is consistent with some previous reports^49,50^, but not all^45^. The increased expression of Nox4 facilitates vascular collagen synthesis and leads to arterial stiffness^49,51^, which may explain the association with abnormal blood pressure. However, the relative contribution of Nox4 expression in ECs and SMCs to the pathogenesis of hypertension requires further research. Overall, our findings suggest that the pathogenesis of hypertension mainly involves ECs, SMCs and fibroblasts, which are the preferred cell subsets for the treatment of hypertension.

Our study has limitations in the following aspects. First, the pathogenesis of hypertension involves many factors, and diverse animal models have been developed to study this disease, including spontaneously hypertensive rats, obesity-related hypertension, deoxycorticosterone acetate-salt hypertension, renovascular hypertension, obesity-induced hypertension, and others. Our results are mainly aimed at high-salt-induced hypertension, which may not be completely consistent with other hypertension models. Second, in this study, we used an enzyme solution to digest aortic cells; each cell type has a different sensitivity to enzymes, so the digestion rate of each cell type is not equal, which might introduce a systematic bias in the study. Spatial transcriptomics sequencing provides the possibility to solve this issue. Last, to avoid the effect of sex-dependent factors on the composition of vascular cells, we only used male mice^52^. To clarify the differences in the gene expression profiles of hypertensive vascular cells between the sexes^52^, further work is needed. In summary, we revealed the response signatures of cell subsets (ECs and SMCs) and intercellular communication during the progression of high-salt-induced hypertension and combined scRNA-seq data with GWAS data to identify potential targets for the treatment of hypertension for specific cell types. These results could contribute to a better understanding of the pathogenesis of hypertension and may contribute to the development of new treatment options.

## Supplementary information

Supplemental material.

## Data Availability

All RNA sequencing data generated from these studies are publicly available in the NCBI SRA: PRJNA489757/PRJNA755351. The computer code used for this study is available from the authors upon request.
