# Supplementary material for: Single-cell analysis of salt-induced hypertensive mouse aortae reveals cellular heterogeneity and state changes
Source: Exp Mol Med. 2021 Dec 3;53(12):1866–76. doi: 10.1038/s12276-021-00704-w (PMC8741768; doi:10.1038/s12276-021-00704-w)
Supplement: Supplementary file 1 — Supplemental material. [file 12276_2021_704_MOESM1_ESM.pdf]

SUPPLEMENTAL MATERIAL

Single-cell analysis of salt-induced hypertensive mouse aorta reveals cellular heterogeneity and state changes

Ka Zhang[#,1], Hao Kan[#,1], Aiqin Mao[#,1], Li Geng[1], Xin Ma*[,1]

[1]Wuxi School of Medicine, Jiangnan University, Wuxi, China

[#]These authors contributed equally to this study.
* Correspondence author

Supplemental Table 1: EC subpopulation features (or functions) gene lists

Inflammatory response to wounding	Lymph vessel development	Migration					Lipid transport
Grn	Nr2f2	Ephb4	Cyp1b1	Vash1	Card10	Lgals8	Cd36
Ager	Foxc1	Mef2c	Plcg1	Dcn	Itgb3	Cxcl13	Mfsd2a
Alox5	Vegfc	Tgfbr1	Hmox1	Clec14a	Adgra2	Adam17	Apoe
	Syk	Nus1	Tnf	Zc3h12a	Gata2	Efnb2	Slc27a4
	Lgals8	Pik3c2a	Srpx2	Vegfa	Tdgf1	Try4	Fabp5
	Foxc2	Smoc2	Sox18	Sp1	Bmper	Try5	Slc27a1
	Efnb2	Stat5a	Nr4a1	Pdcd6	Rhoa	Try10	
	Ppp3cb	Rhob	Prkca	Prkx	Rras	Prss3	
	Tmem204	Rock2	Ceacam10	Efna1	Nr2e1	Gm5771	
	Tbx1	Mmrn2	Ceacam2	Gpi1	Hrg	Gm10334	
	Heg1	Atp2b4	Ceacam1	Pten	Ptk2b	Prss1	
	Ptpn14	Fgfr1	Stc1	Csnk2b	Rhoj	Prss2	
	Flt4	Pxn	Egr3	Scarb1	Gm42906	Adtrp	
	Ccbe1	Grn	Apoa1	Hif1a	Stard13	Gadd45a	
	Pkd1	Atp5b	Loxl2	Ets1	Dpp4	Adgrb1	
	Pdpn	Tgfb1	Acvrl1	Aamp	Notch1	Fgf4	
	Lgals12	Hmgb1	P2rx4	Gata3	Gpx1	Hdac7	
	Epha2	Fgf1	Ptgs2	Zfp580	Anxa3	Emp2	
	Bmpr2	Gpld1	Robo1	Angpt1	Myh9	Gdf2	
	Prox1	Cdh13	Alox12	Edn1	Dnaja4	Meox2	
	Sox18	Angpt2	Sash1	Map2k3	Plk2	Bmp10	
	Svep1	Wnt7a	Nrp1	Pdcd10	Slit2	Rin2	
	Acvrl1	Prcp	Akt1	Met	Apoh	Pdgfb	
	Vash1	Wnt5a	Mecp2	Agtr2	Tmsb4x	Rgcc	
	Vegfa	Ptn	Fgf16	Map3k3	Gm49510	Itgb2	
	Acvr2b	Hspb1	Nrp2	Gm49337	Sh3bp1	Itgb2l	
		Fbxw7	Akt3	Paxip1	Thbs1	Ptprm	
		Tnfsf12	Amot	Tek	Agt	Id1	
		Dll4	Epha2	Lgmn	Svbp	Nf1	
		Hdac9	Dab2ip	Grem1	Jcad	Atoh8	
		Sema4a	Pdpk1	Fut1	Ptk2	Prkd1	
		S100a2	Adamts9	Cib1	Tbxa2r	Lgals12	
		Pik3ca	Flt4	Kdr	Map2k5	Srf	
		Coro1b	Fgf2	Spred1	Glul	Rab13	
		Cdh5	Pecam1	Prox1	Bcar1	Scg2	
		Sema5a	Anxa1	Fap	Ccbe1	Plekhg5	
		Jup	Bmpr2	Lpxn	Pparg	Gipc1	
		Calr	Apoe	Sparc	Itgb1	Ccn3	
		Sirt1	Cd40	Foxc2	Amotl1	Abl1	

		Fgf18	Plxnd1	Angpt4	Bcas3	Hdac5	
		Fgfbp1	Atp5a1	Itgb1bp1	Klf4	Nos3	
		Krit1	Prkd2	Plpp3	Vegfc	Ptp4a3	
		Nfe2l2	Serpinf1	Bmp4	Pik3r3	Nr2f2	
		Emc10					

Contractile	Collagen	MHC protein complex	Proteoglycan	Differentiation	ROS-related enzymes	Proliferation
Actg2	Col1a2	B2m	Fmod	Ccm2	Nox1	Itga4
Tpm2	Col4a2	B2m	Prelp	Dll1	Nox4	Mef2c
Myo1d	Col27a1	Cd74	Srgn	Rbpj	Nox2	Stat3
Myh11	Col5a2	Cd74	Gpc3	Hey2	Nox3	Ccl2
Tpm4	Col8a1	H2-Aa	Gpc4	Tmem100	Nox5	Hmgb1
Actb	Col4a4	H2-Aa	Agrn	Hey1	Sod1	Fgfr1
Actg1	Col4a3	H2-Aa	Gpc1	Foxj2	Sod2	Pdpk1
Myo19	Col5a1	H2-Ab1	Sdc2	Nrp1	Sod3	Sirt6
Acta2	Col4a1	H2-Ab1	Aspn	Notch1	Ptgs1	Adam17
Actc1	Col3a1	H2-D1	Omd		Ptgs2	Ghsr
Tnnt3	Col14a1	H2-DMa	Ogn		Cybb	Col4a3
Myo18a	Col9a2	H2-DMb1	Lum		Xdh	Plcg1
Myo9a	Col6a1	H2-DMb2	Sdc3			Igf2
Myo1e	Col16a1	H2-Eb1	Smc3			Sp1
Myh9	Col28a1	H2-K1	Cd44			Apln
Myo1f	Col5a3	H2-L	Bgn			Fgf2
Myo5c	Col15a1	H2-M3	Sdc4			Akt3
Myo9b	Col18a1	H2-Oa	Acan			Pparg
Myo6	Col6a2	H2-Ob	Hspg2			Ghrl
Myo15b	Col25a1	H2-Q1	Vcan			Flt1
Myh10	Col6a3	H2-Q2	Podn			
Myo1c	Col8a2	H2-Q4	Cspg4			
Tpm3	Col12a1	H2-Q6	Tgfbr3			
Tpm3-rs7	Col13a1	H2-Q7	Gpc6			
Myo1b	Col1a1	H2-Q9	Dcn			
Myo5a	Col4a5	H2-Q10				
Myo10		H2-T23				
Tpm1		H2-T23				
		Mr1				

List of genes used to define EC subpopulation features (or functions) scores

Supplemental Table 2: contractile SMC markers gene list

contractile SMC markers
Acta2
Myh11
Myh9
Myh7
Myh6
Myh13
Myh10
Myh2
Myh4
Myh14
Myh3
Myh1
Tagln
Smtn
Mylk
Vcl
Des

List of genes used to define contractile SMC.

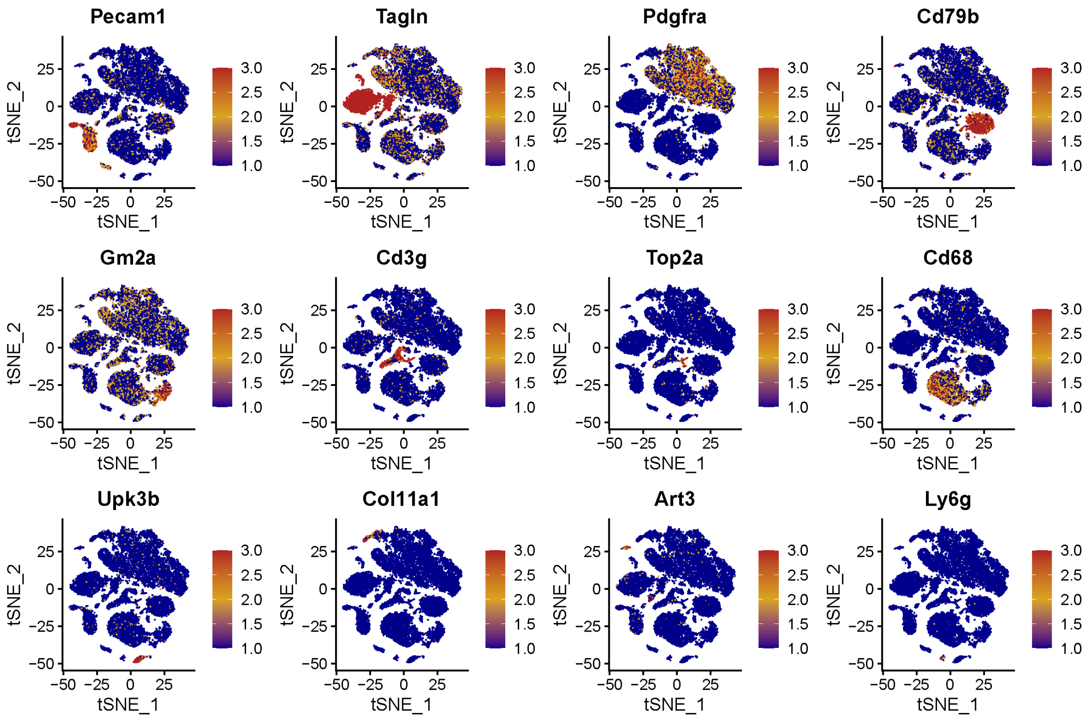

Supplemental Figure 1 Expression of genes correlated with major cell types onto a t-SNE plot

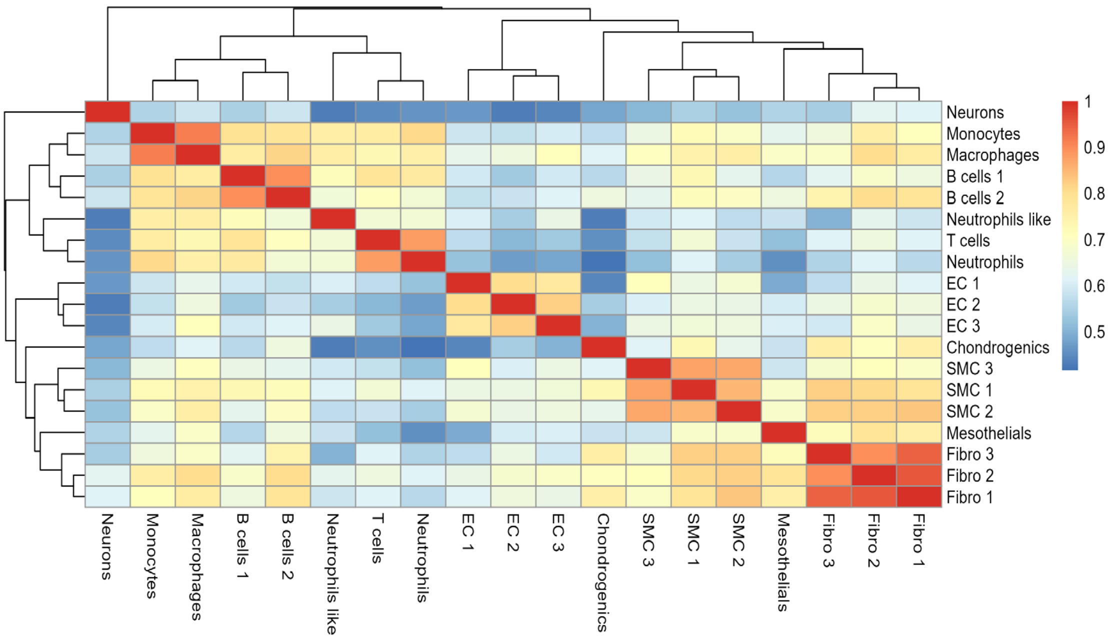

Supplemental Figure 2 Correlation of 19 cell types in the mouse aortic arch

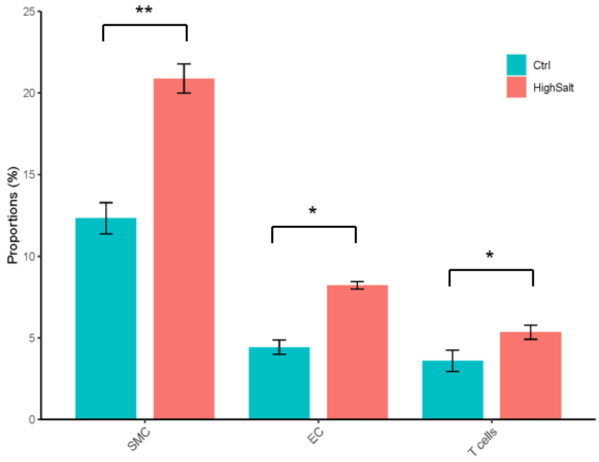

Supplemental Figure 3 The percentages of the SMC, EC and T cells population from control and high-salt diet mice. Data are represented as the mean ± SEM. **P < 0.01 and *P < 0.05 by Student's t test. n=4 control vs. n=3 high-salt diet mice.

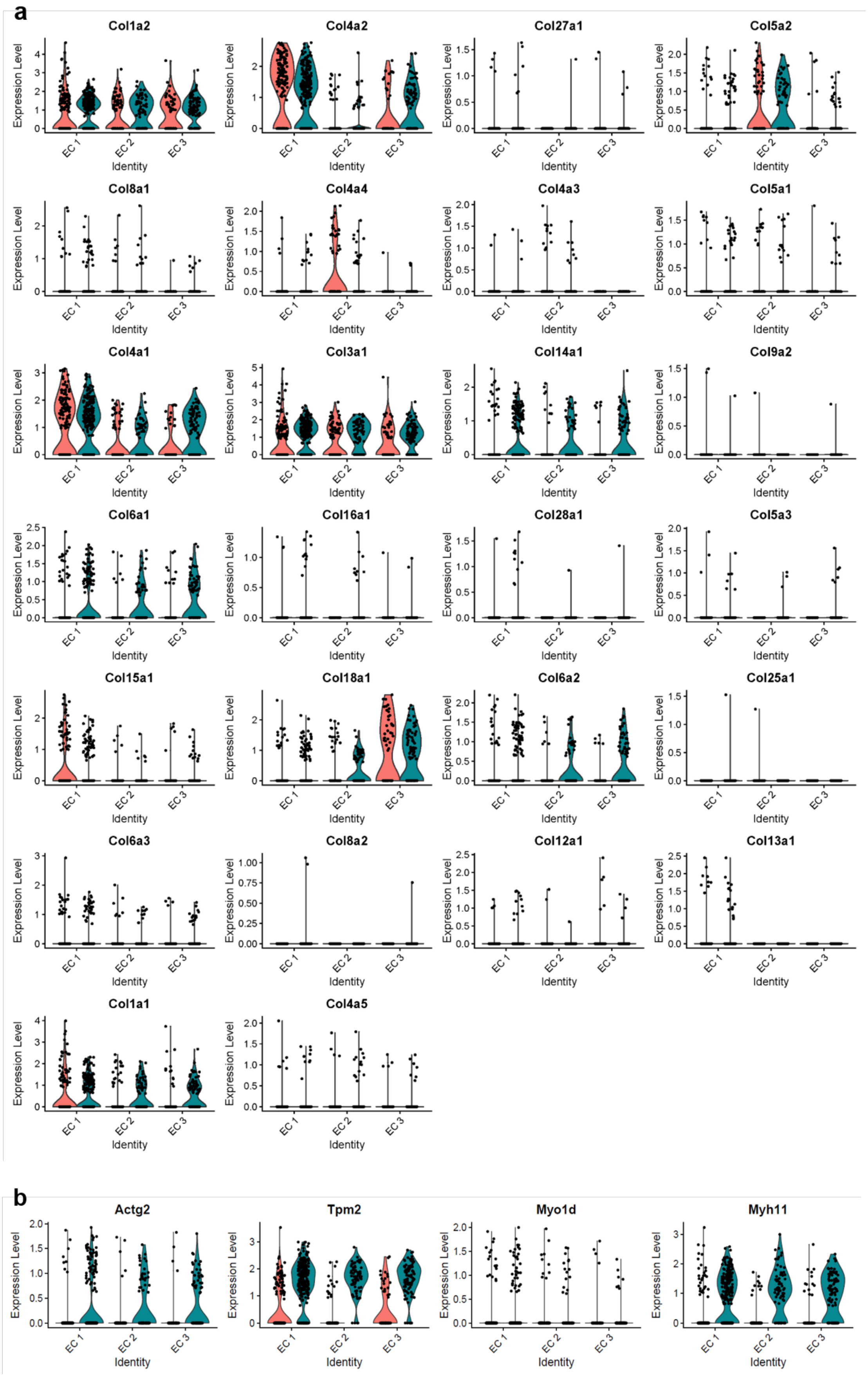
a
Col1a2
Col4a2
Col27a1
Col5a2
Col8a1
Col4a4
Col4a3
Col5a1
Col4a1
Col3a1
Col14a1
Col9a2
Col6a1
Col16a1
Col28a1
Col5a3
Col15a1
Col18a1
Col6a2
Col25a1
Col6a3
Col8a2
Col12a1
Col13a1
Col1a1
Col4a5
b
Actg2
Tpm2
Myo1d
Myh11
Expression Level
Identity
EC 1
EC 2
EC 3

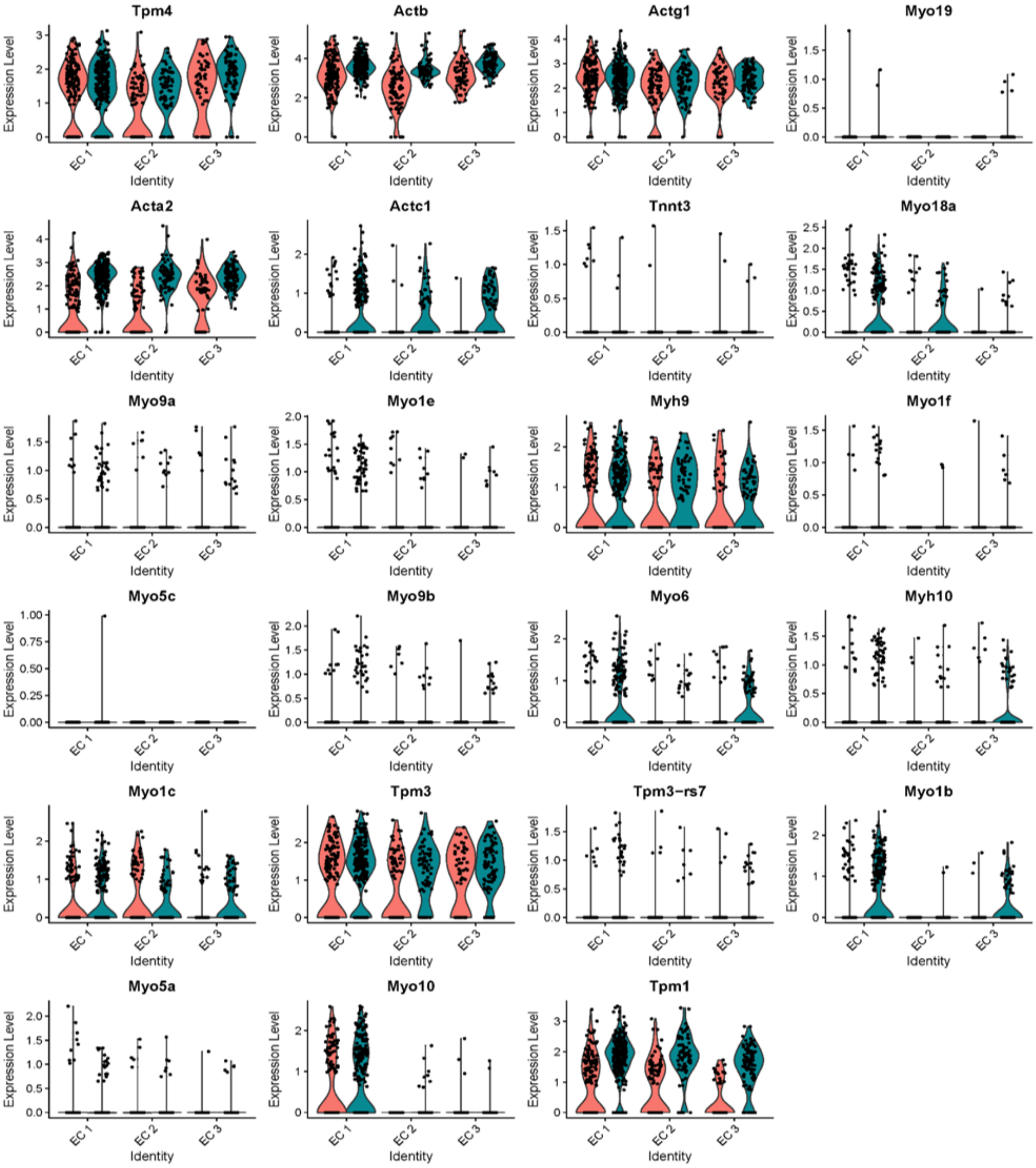
Tpm4
Actb
Actg1
Myo19
Acta2
Actc1
Tnnt3
Myo18a
Myo9a
Myo1e
Myh9
Myo1f
Myo5c
Myo9b
Myo6
Myh10
Myo1c
Tpm3
Tpm3-rs7
Myo1b
Myo5a
Myo10
Tpm1
Expression Level
Identity
EC 1
EC 2
EC 3

C

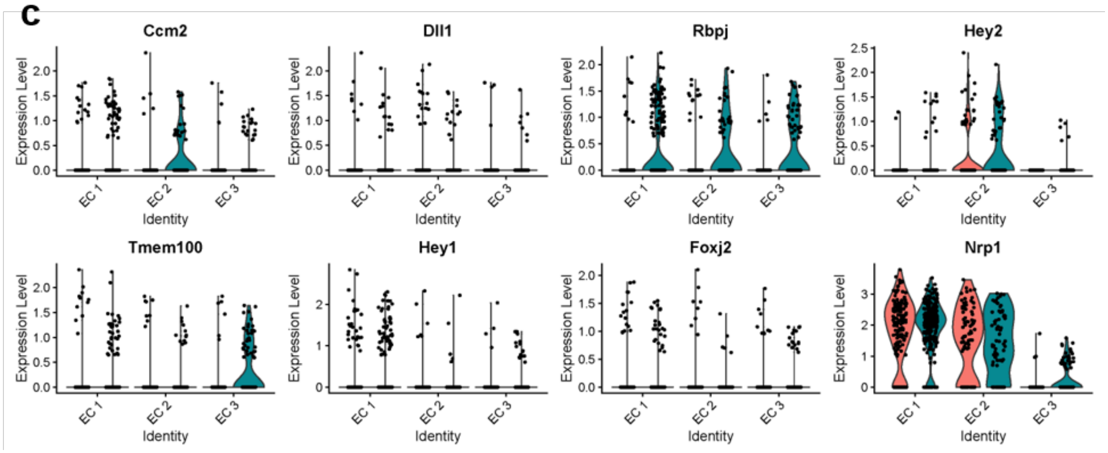
Ccm2
Dll1
Rbpj
Hey2
Tmem100
Hey1
Foxj2
Nrp1
Expression Level
Identity
EC 1
EC 2
EC 3

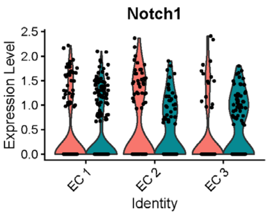
Notch1
Expression Level
EC 1
EC 2
EC 3
Identity

d

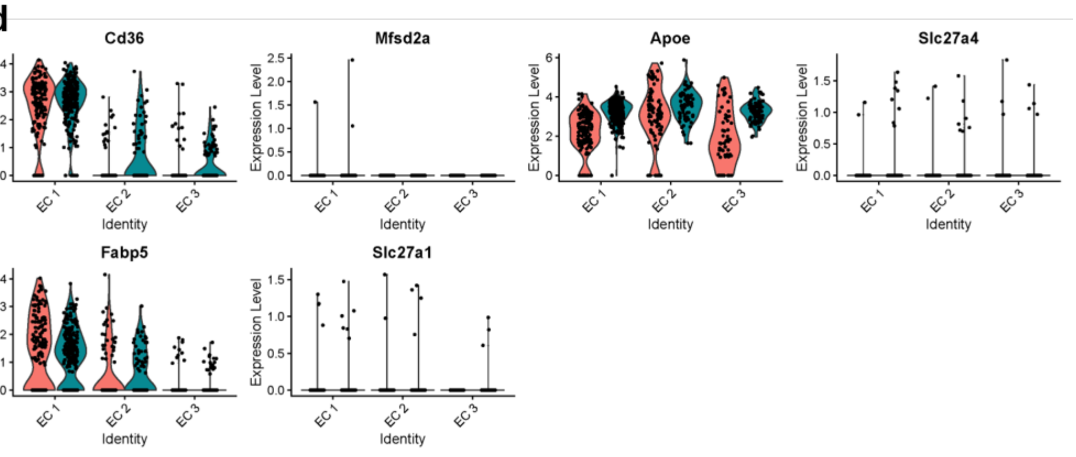
Cd36
Mfsd2a
Apoe
Slc27a4
Fabp5
Slc27a1
Expression Level
EC 1
EC 2
EC 3
Identity

e

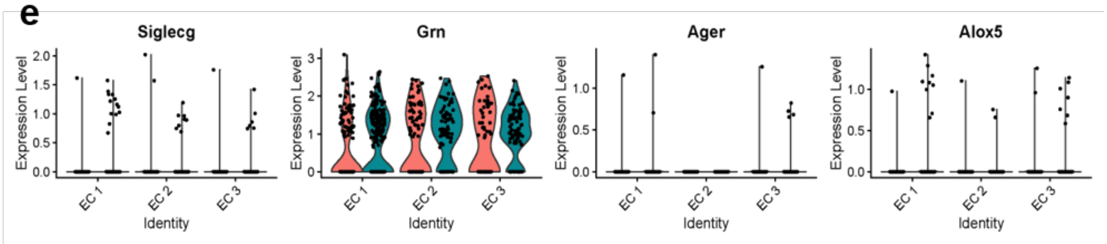
Siglecg
Grn
Ager
Alox5
Expression Level
EC 1
EC 2
EC 3
Identity

f

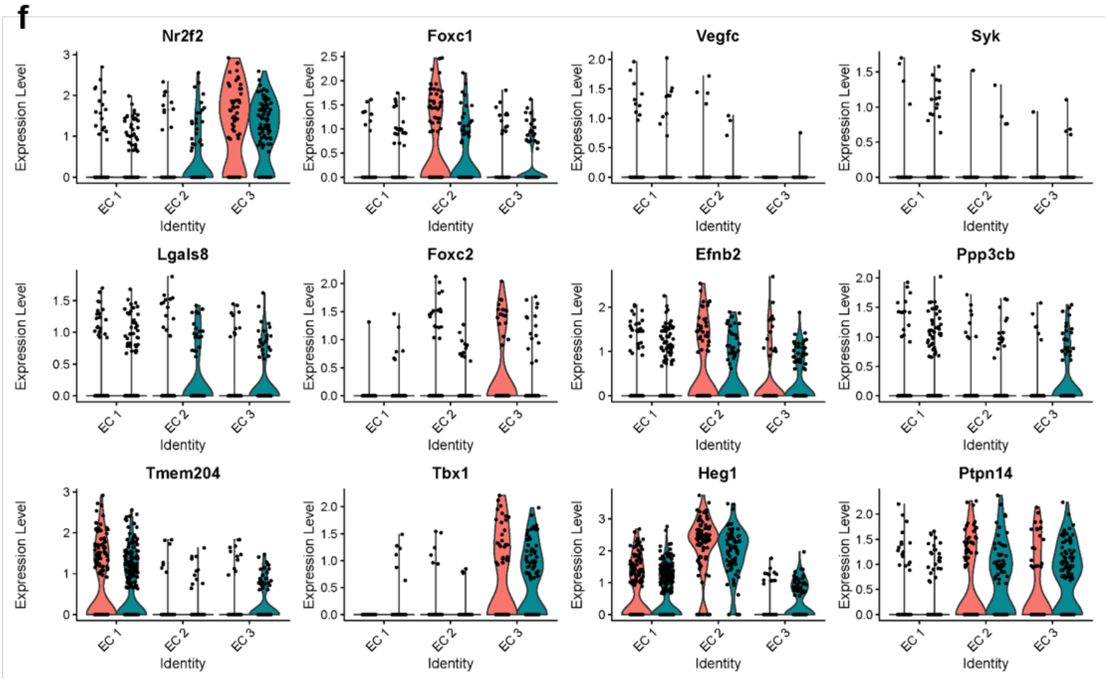
Nr2f2
Foxc1
Vegfc
Syk
Lgals8
Foxc2
Efnb2
Ppp3cb
Tmem204
Tbx1
Heg1
Ptpn14
Expression Level
EC 1
EC 2
EC 3
Identity

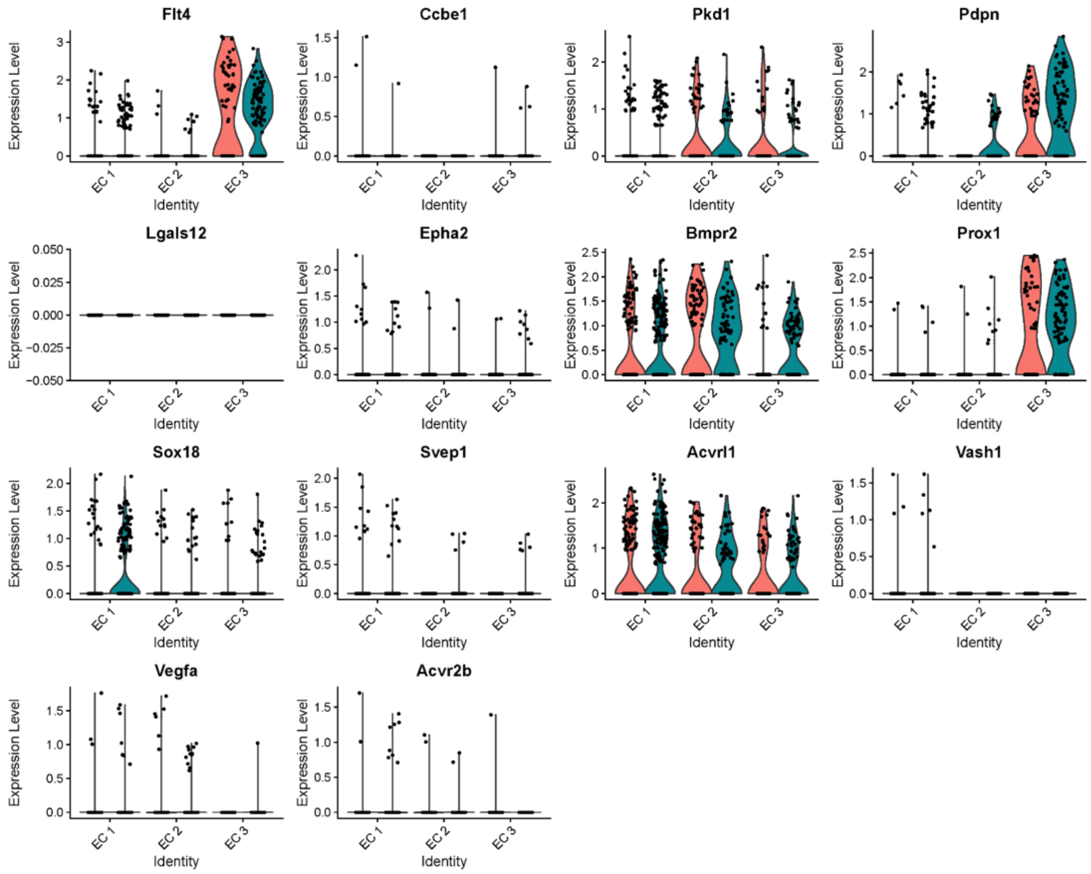

Flt4
Ccbe1
Pkd1
Pdpn
Lgals12
Epha2
Bmpr2
Prox1
Sox18
Svep1
Acvrl1
Vash1
Vegfa
Acvr2b
Expression Level
Identity
EC 1
EC 2
EC 3

g

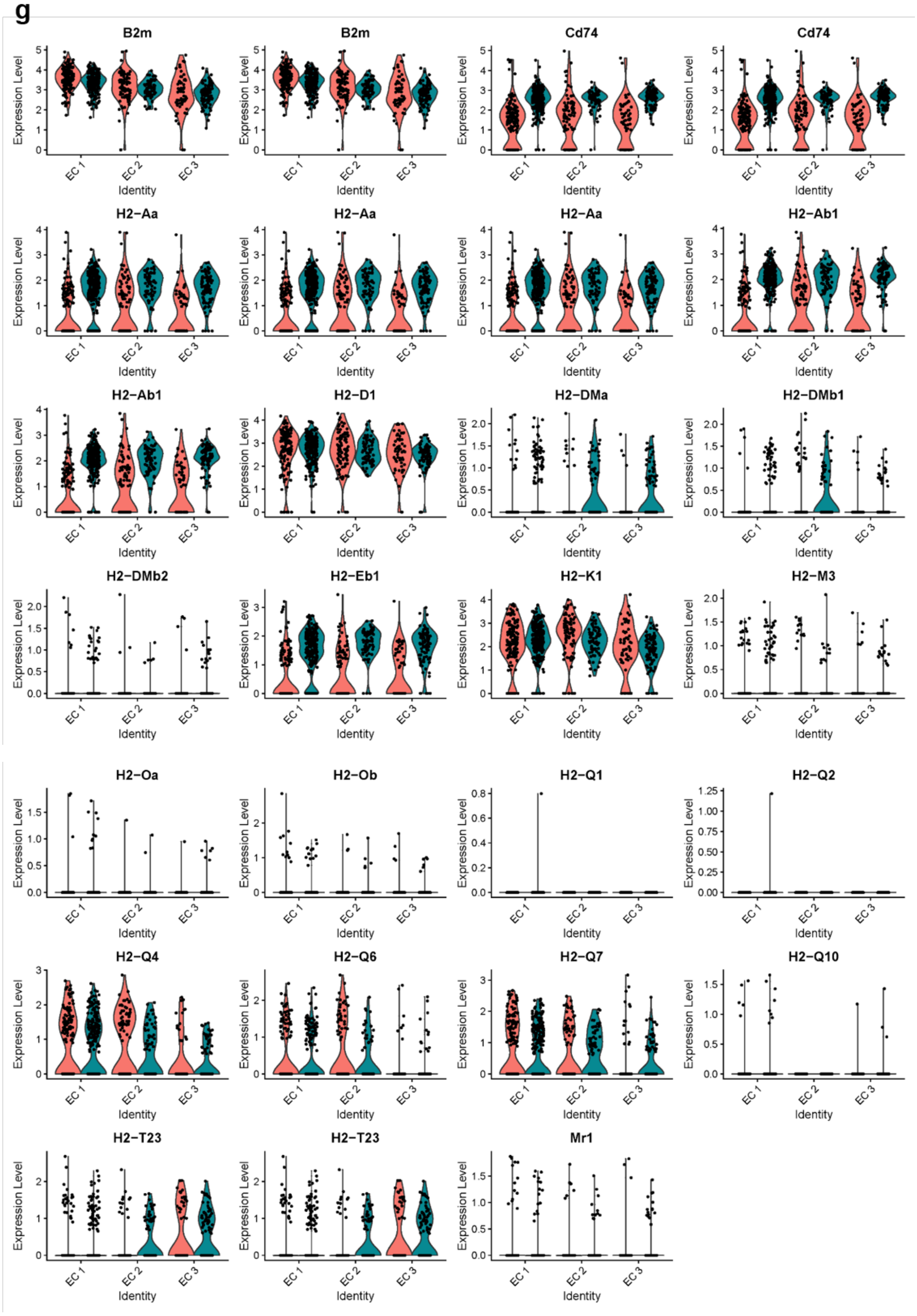
B2m
B2m
Cd74
Cd74
H2−Aa
H2−Aa
H2−Aa
H2−Ab1
H2−Ab1
H2−D1
H2−DMa
H2−DMb1
H2−DMb2
H2−Eb1
H2−K1
H2−M3
H2−Oa
H2−Ob
H2−Q1
H2−Q2
H2−Q4
H2−Q6
H2−Q7
H2−Q10
H2−T23
H2−T23
Mr1
Expression Level
Identity
EC 1
EC 2
EC 3

h

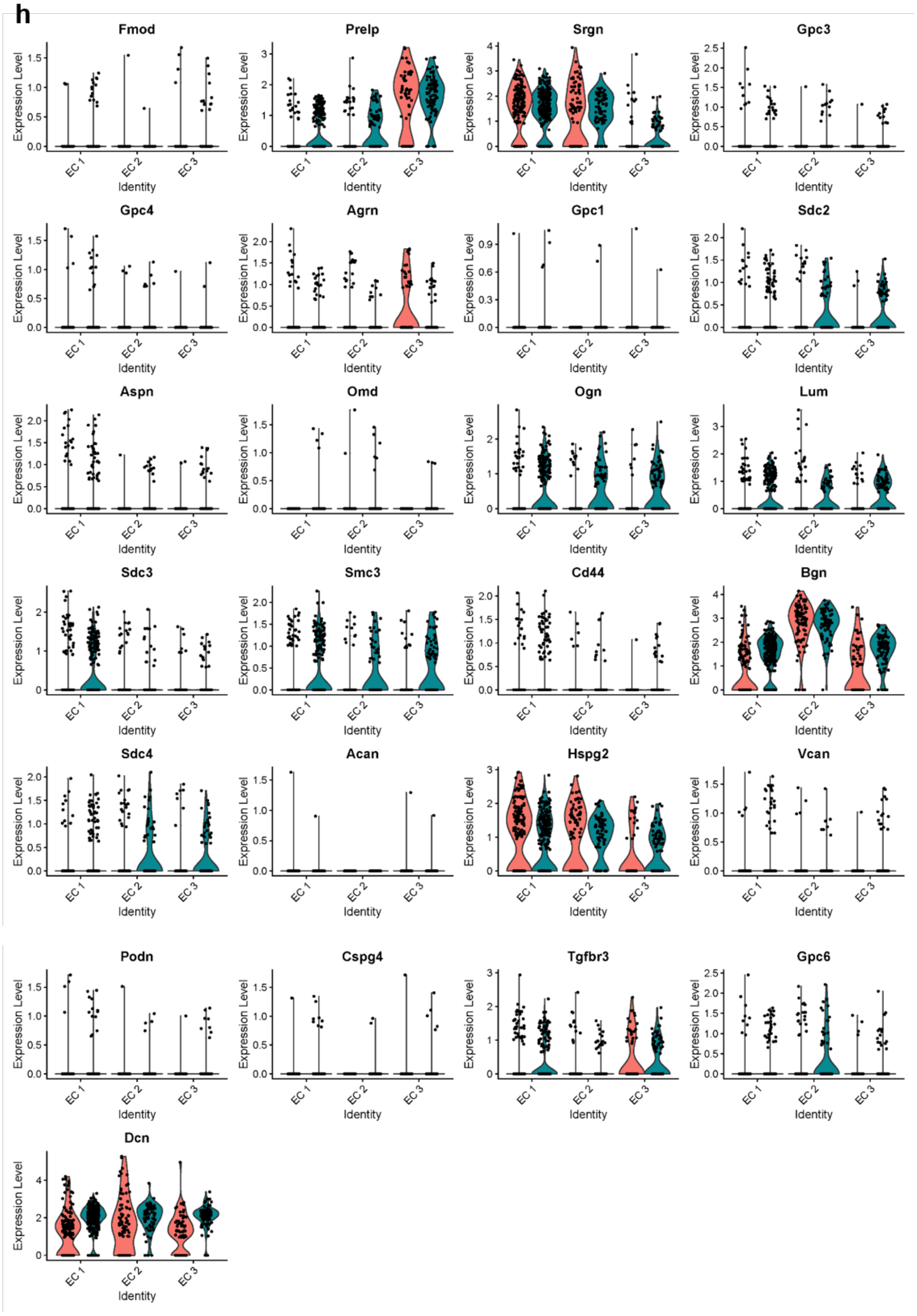
Fmod
Prelp
Srgn
Gpc3
Gpc4
Agrn
Gpc1
Sdc2
Aspn
Omd
Ogn
Lum
Sdc3
Smc3
Cd44
Bgn
Sdc4
Acan
Hspg2
Vcan
Podn
Cspg4
Tgfbr3
Gpc6
Dcn
Expression Level
EC 1
EC 2
EC 3
Identity

i

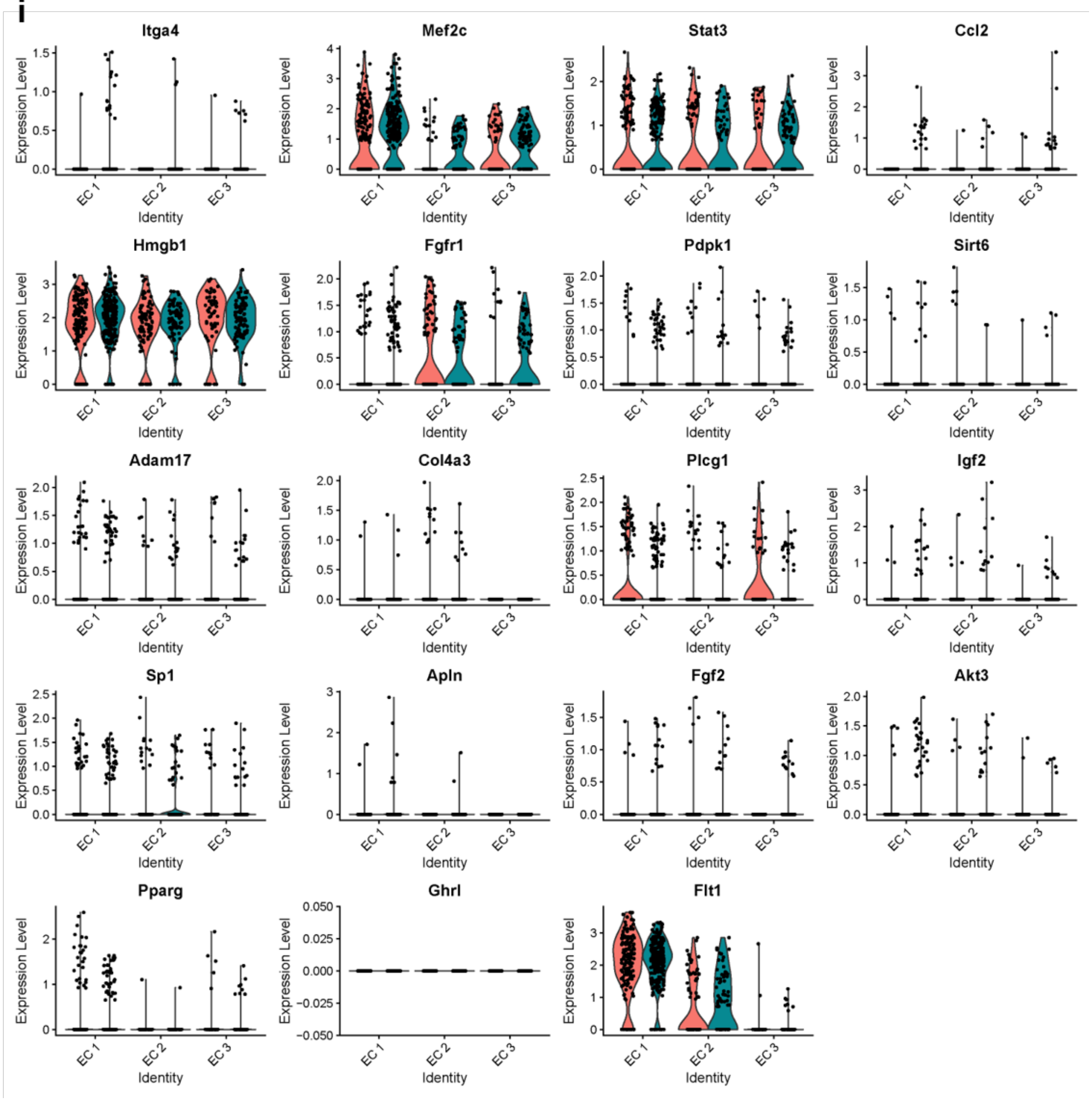
Itga4
Mef2c
Stat3
Ccl2
Hmgb1
Fgfr1
Pdpk1
Sirt6
Adam17
Col4a3
Plcg1
Igf2
Sp1
Apln
Fgf2
Akt3
Pparg
Ghrl
Flt1
Expression Level
Identity
EC 1
EC 2
EC 3

j

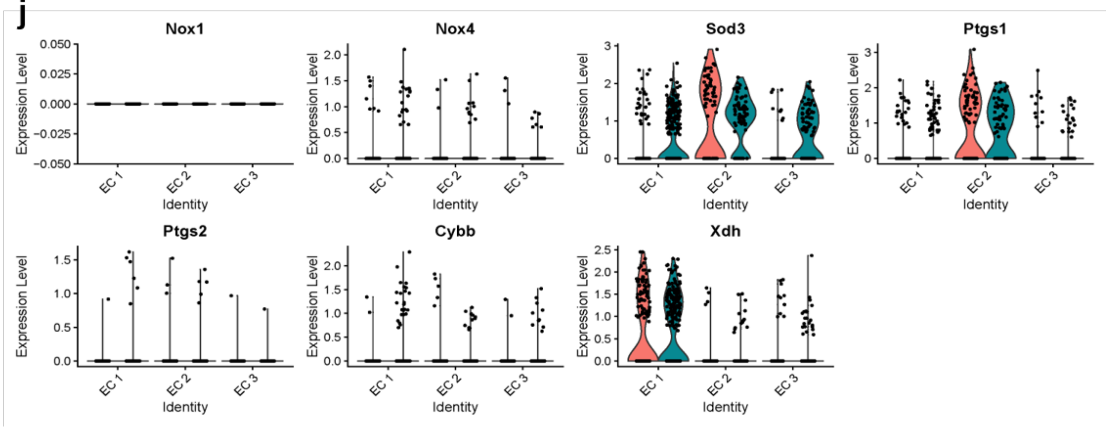
Nox1
Nox4
Sod3
Ptgs1
Ptgs2
Cybb
Xdh
Expression Level
Identity
EC 1
EC 2
EC 3

k

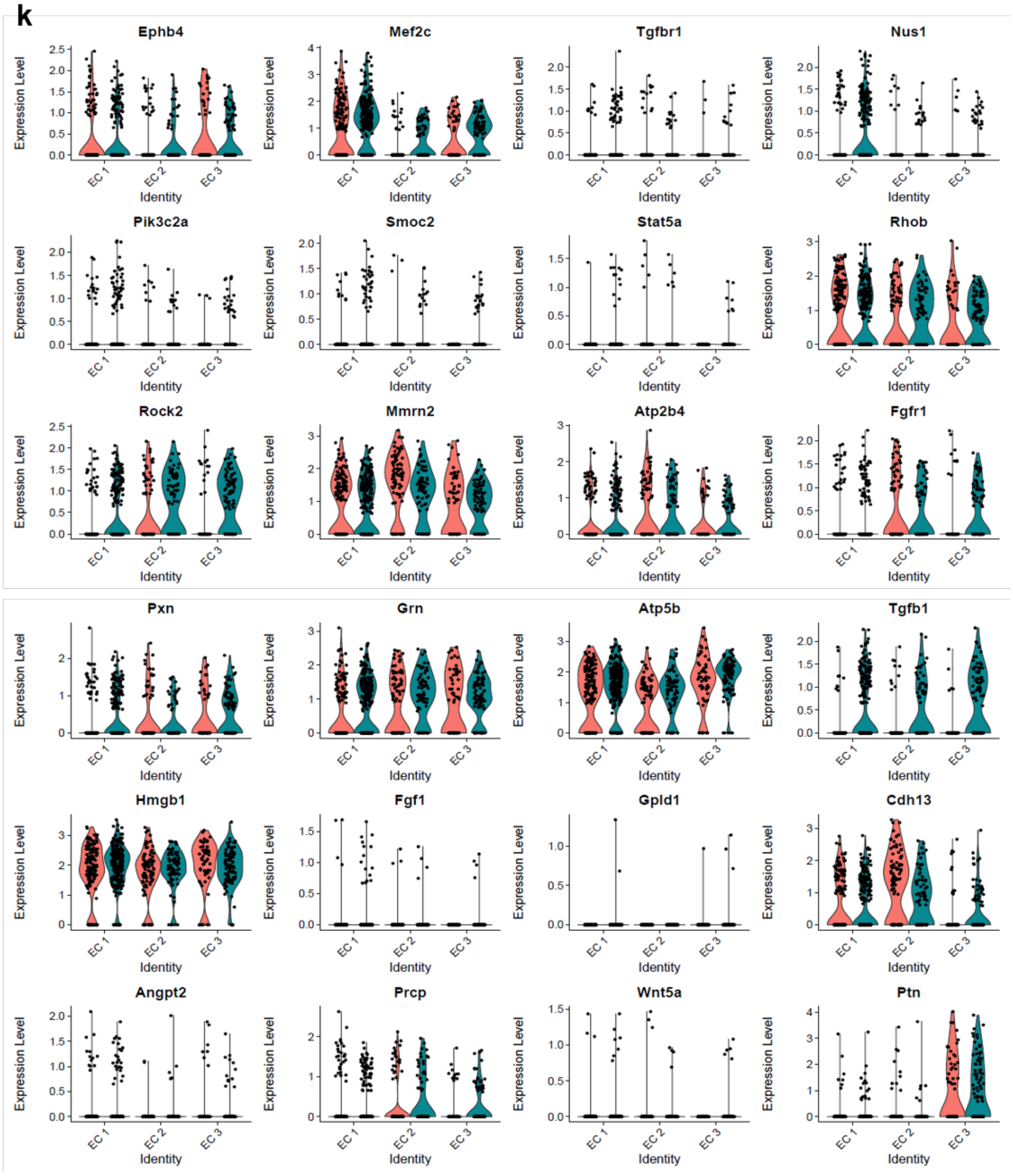
Ephb4
Mef2c
Tgfbr1
Nus1
Pik3c2a
Smoc2
Stat5a
Rhob
Rock2
Mmrn2
Atp2b4
Fgfr1
Pxn
Grn
Atp5b
Tgfb1
Hmgb1
Fgf1
Gpld1
Cdh13
Angpt2
Prcp
Wnt5a
Ptn
Expression Level
Identity
EC 1
EC 2
EC 3

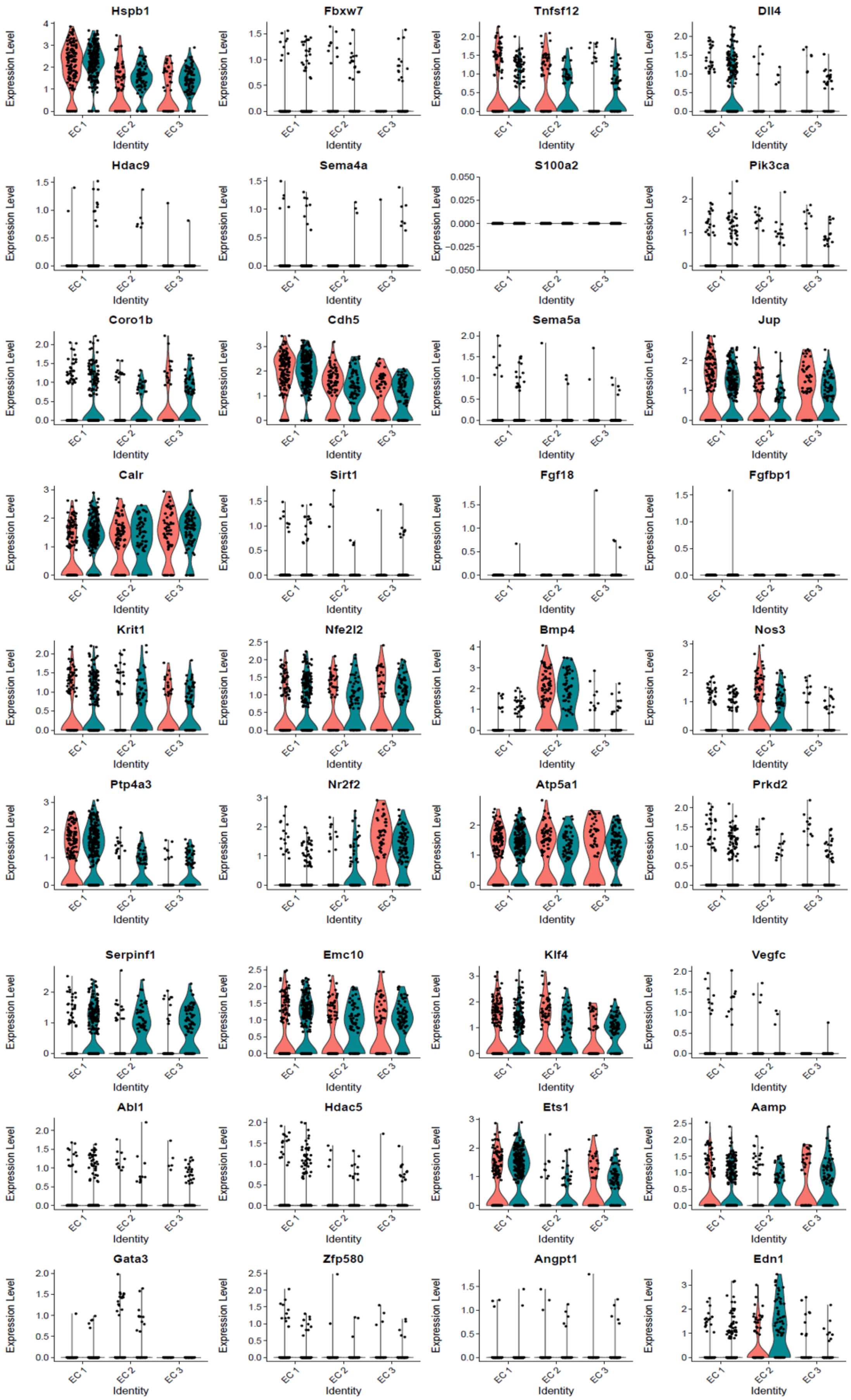

Hspb1
Fbxw7
Tnfsf12
Dll4
Hdac9
Sema4a
S100a2
Pik3ca
Coro1b
Cdh5
Sema5a
Jup
Calr
Sirt1
Fgf18
Fgfbp1
Krit1
Nfe2l2
Bmp4
Nos3
Ptp4a3
Nr2f2
Atp5a1
Prkd2
Serpinf1
Emc10
Klf4
Vegfc
Abl1
Hdac5
Ets1
Aamp
Gata3
Zfp580
Angpt1
Edn1
Expression Level
Identity
EC 1
EC 2
EC 3

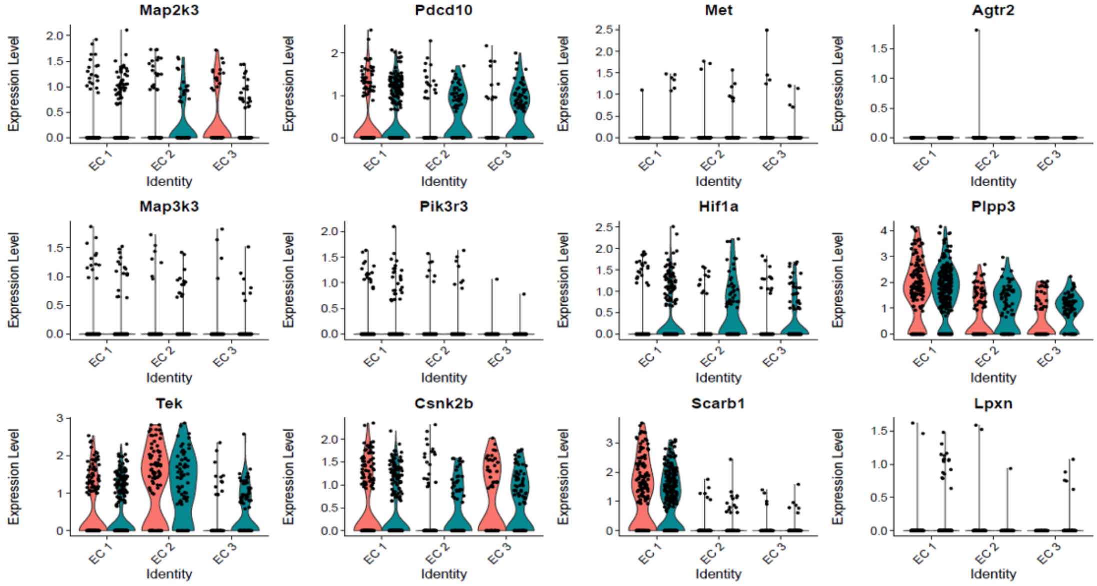
Map2k3
Pdcd10
Met
Agtr2
Map3k3
Pik3r3
Hif1a
Plpp3
Tek
Csnk2b
Scarb1
Lpxn
Expression Level
Identity
EC 1
EC 2
EC 3

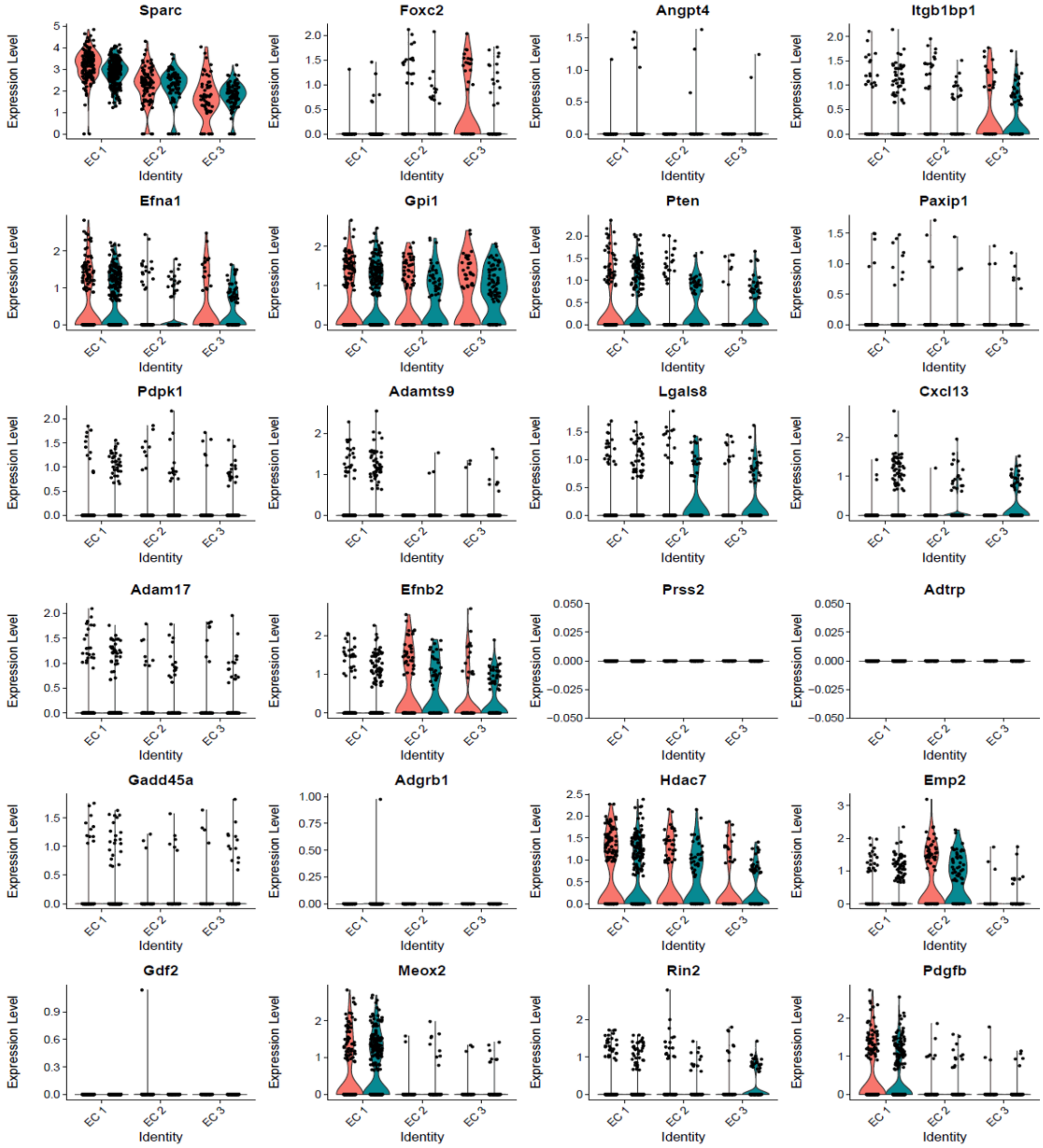
Sparc
Foxc2
Angpt4
Itgb1bp1
Efna1
Gpi1
Pten
Paxip1
Pdpk1
Adamts9
Lgals8
Cxcl13
Adam17
Efnb2
Prss2
Adtrp
Gadd45a
Adgrb1
Hdac7
Emp2
Gdf2
Meox2
Rin2
Pdgfb
Expression Level
Identity
EC 1
EC 2
EC 3

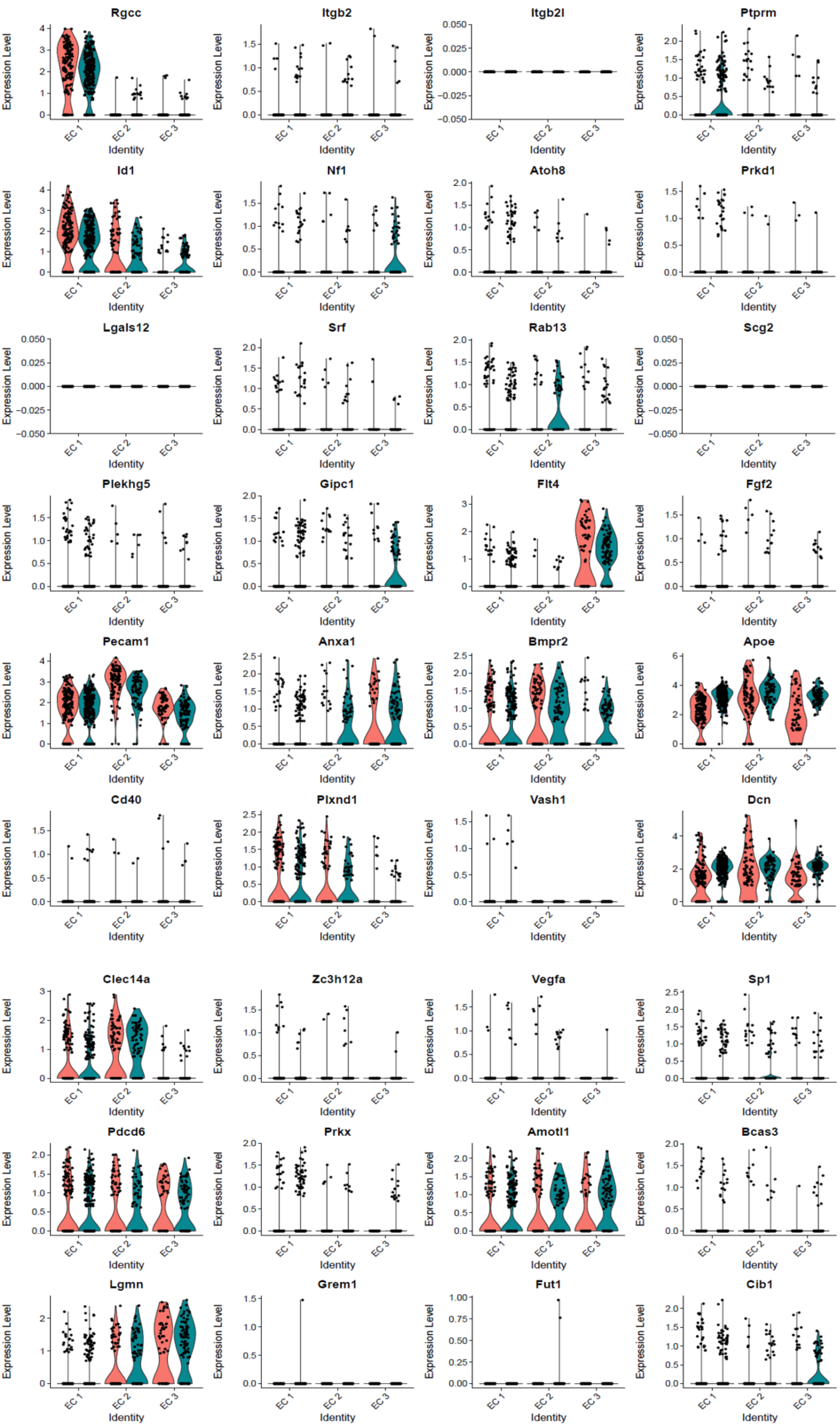
Rgcc
Itgb2
Itgb2l
Ptprm
Id1
Nf1
Atoh8
Prkd1
Lgals12
Srf
Rab13
Scg2
Plekhg5
Gipc1
Flt4
Fgf2
Pecam1
Anxa1
Bmpr2
Apoe
Cd40
Plxnd1
Vash1
Dcn
Clec14a
Zc3h12a
Vegfa
Sp1
Pdcd6
Prkx
Amotl1
Bcas3
Lgmn
Grem1
Fut1
Cib1
Expression Level
Identity
EC 1
EC 2
EC 3

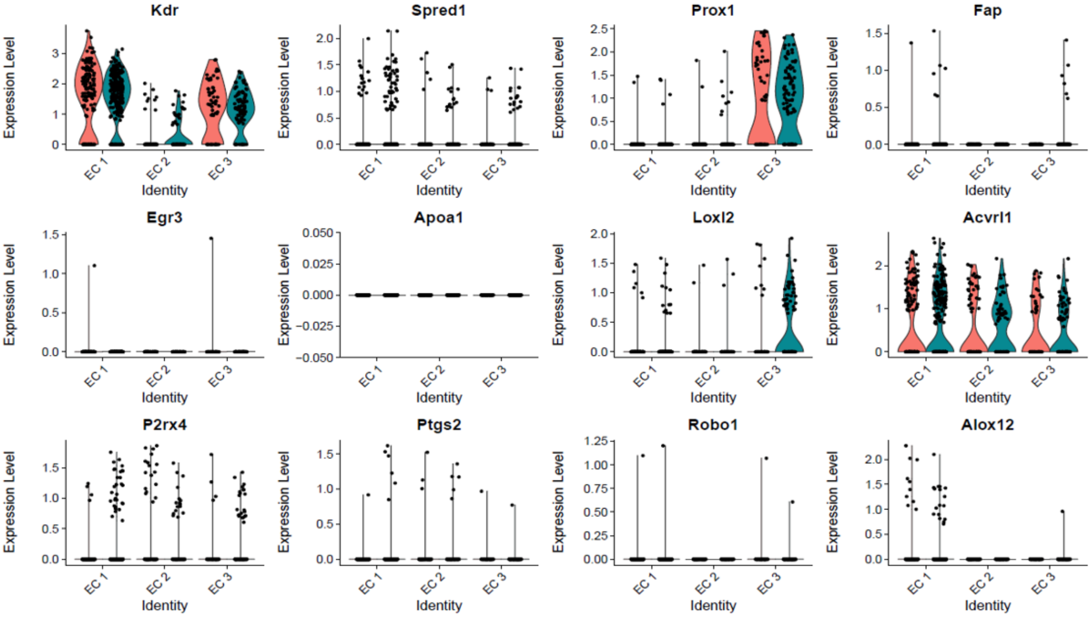
Kdr
Spred1
Prox1
Fap
Egr3
Apoa1
Loxl2
Acvrl1
P2rx4
Ptgs2
Robo1
Alox12
Expression Level
Identity
EC 1
EC 2
EC 3

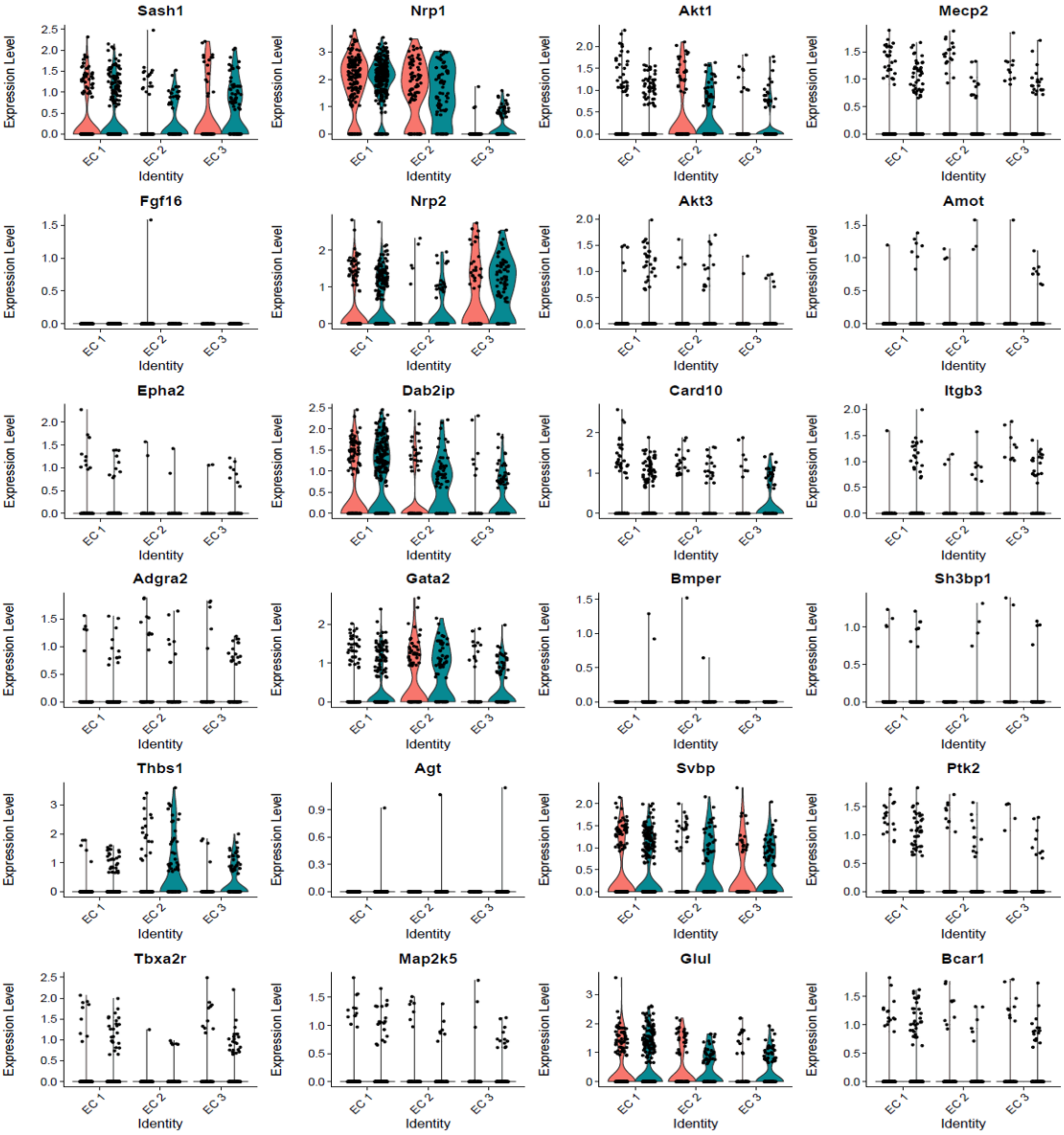
Sash1
Nrp1
Akt1
Mecp2
Fgf16
Nrp2
Akt3
Amot
Epha2
Dab2ip
Card10
Itgb3
Adgra2
Gata2
Bmper
Sh3bp1
Thbs1
Agt
Svbp
Ptk2
Tbxa2r
Map2k5
Glul
Bcar1
Expression Level
Identity
EC 1
EC 2
EC 3

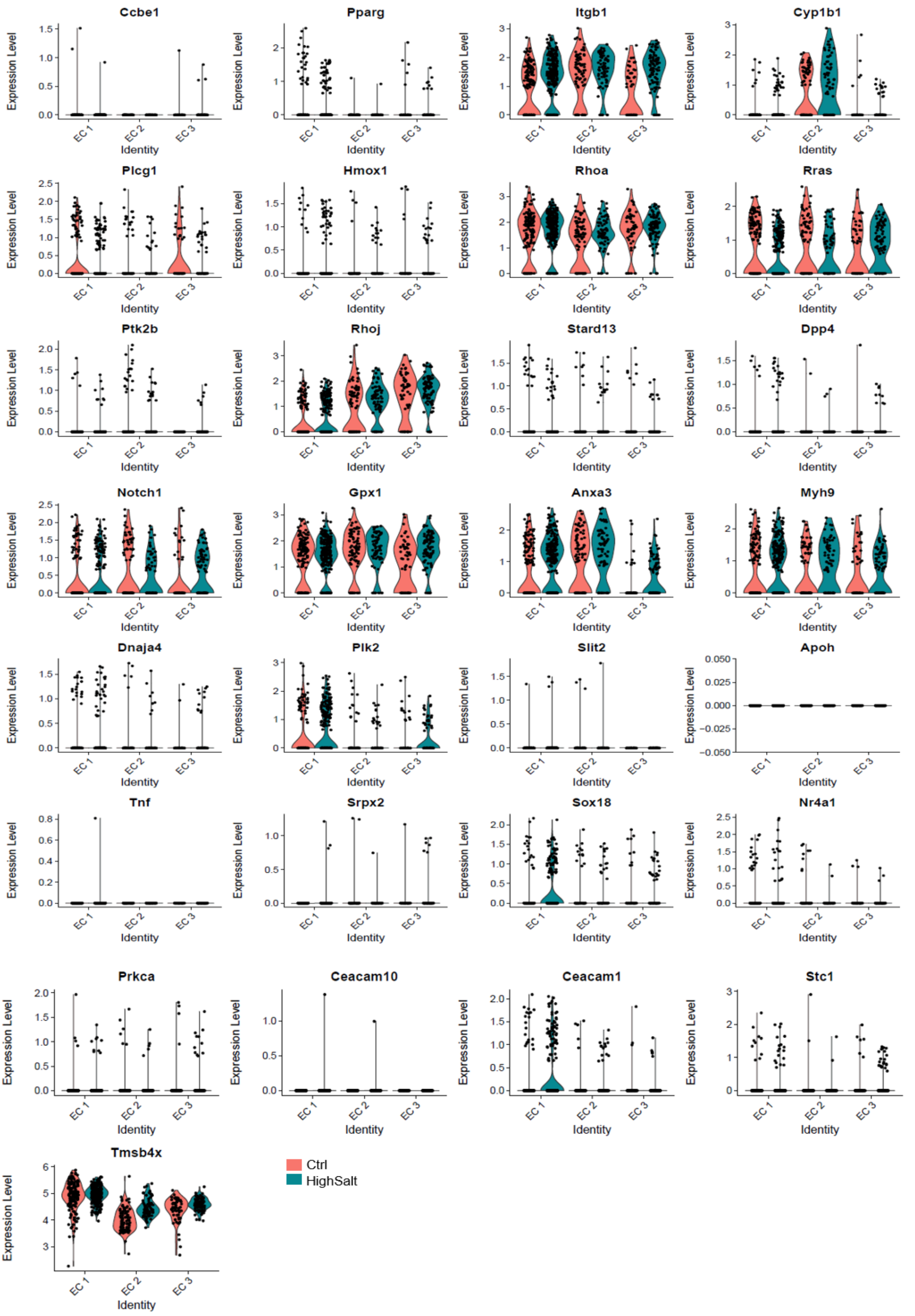

Supplemental Figure 4 Violin plot showing the expression of genes related to endothelial cell features (or functions). a: Collagen, **b:** Contractile, **c:** Differentiation, **d**: Lipid transport, **e:** Inflammatory response to wounding, **f:** Lymph vessel development, **g:** MHC protein complex, **h:** Proteoglycan, **i:** Proliferation, **j:** ROS-related enzymes, **k:** Migration.

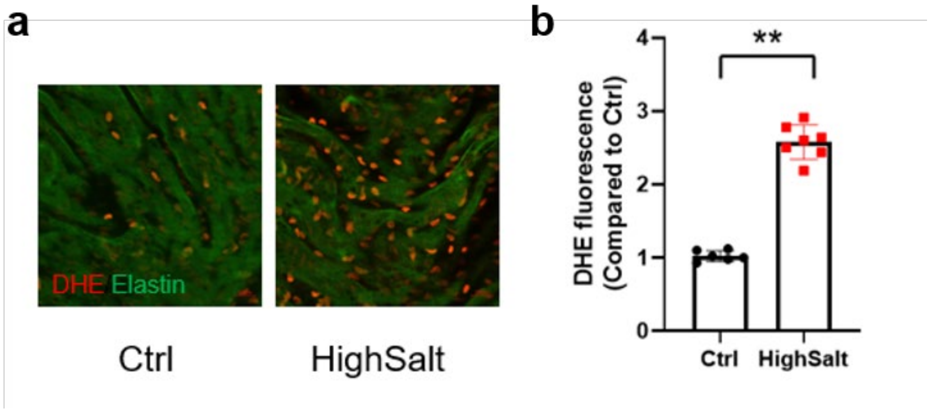

Supplemental Figure 5 High-salt diet enhanced endothelial reactive oxygen species (ROS) production. a (representative images) and **b** (summarized data), ROS measured by DHE in the en face endothelium of aortae from control and High-salt diet mice. Bar: 20μm. Data are represented as the mean ± SEM. **P < 0.01 by Student's t test. n = 6-7.

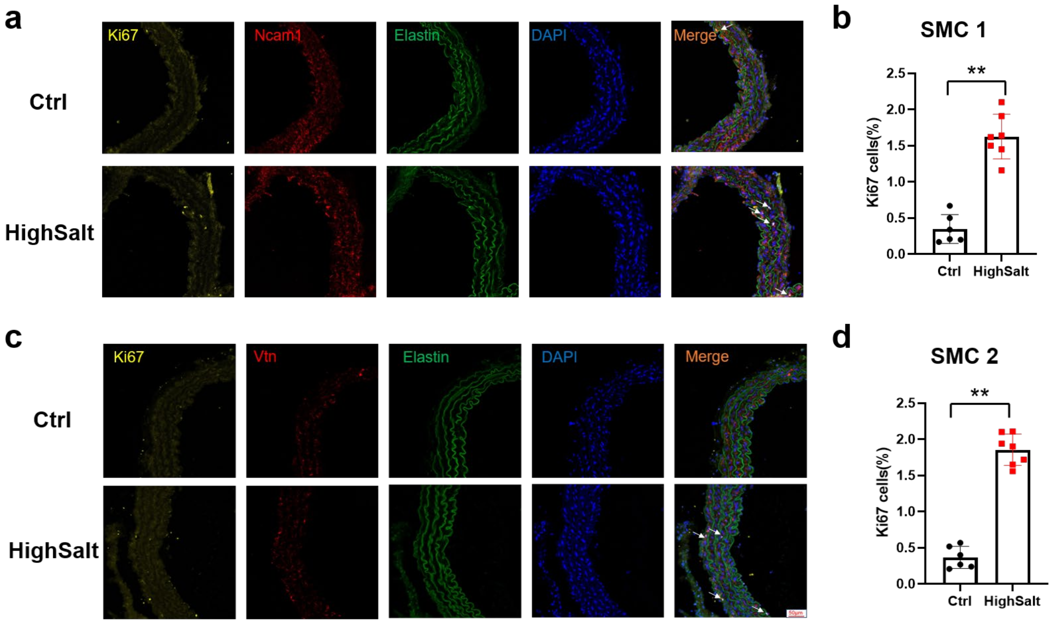

Supplemental Figure 6 Quantification of double-immunostained cells with Ncam1/Vtn (SMC1/SMC2 marker) and Ki67 markers in aortic arch section. a and **c** are representative of immunostaining for Ncam1/Vtn (red, SMC1/SMC2) and Ki67 (yellow). **b** and **d** are summarized data. DAPI detection of nuclei is represented in blue, and green represents elastin autofluorescence. Arrows point to proliferating SMC. Scale bar: 50 μm. Data are represented as the mean ± SEM. **P < 0.01 by Student's t test. n = 6-8.

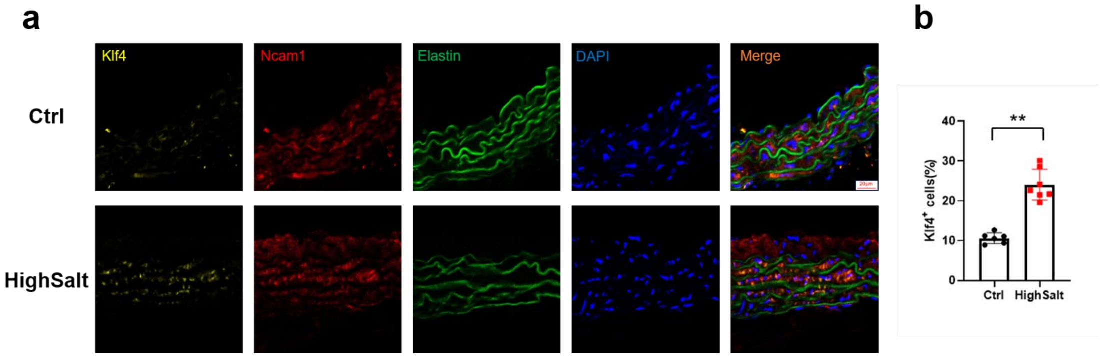

Supplemental Figure 7 High-salt diet induces increased Klf4 expression in SMC **(a)** Representative of immunostaining for Ncam1 (red, SMC1) and Klf4(yellow). (**b**) Summarized data. DAPI detection of nuclei is represented in blue, and green represents elastin autofluorescence; scale bar: 20 μm. Data are represented as the mean ± SEM. **P < 0.01 by Student's t test. n = 6-8.

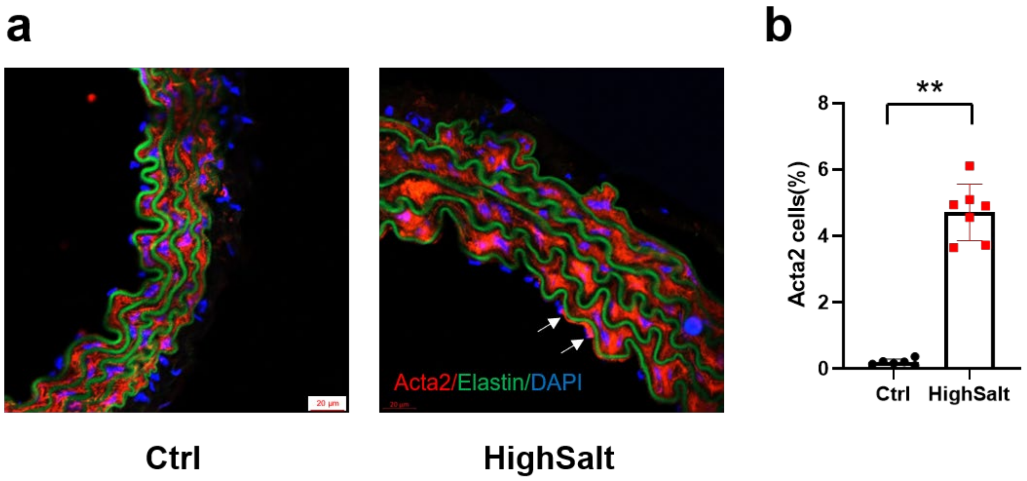

Supplemental Figure 8 Examples of ACTA2 expression in monolayers of cells lining the vasculature on histological sections of the aortic arch in control and high-salt diet mice. a (representative images) and **b** (summarized data). Arrows point to Acta2-expressing endothelial cells. Scale bar: 20 μm**.** Data are represented as the mean ± SEM. **P < 0.01 by Student's t test. n = 6-8.

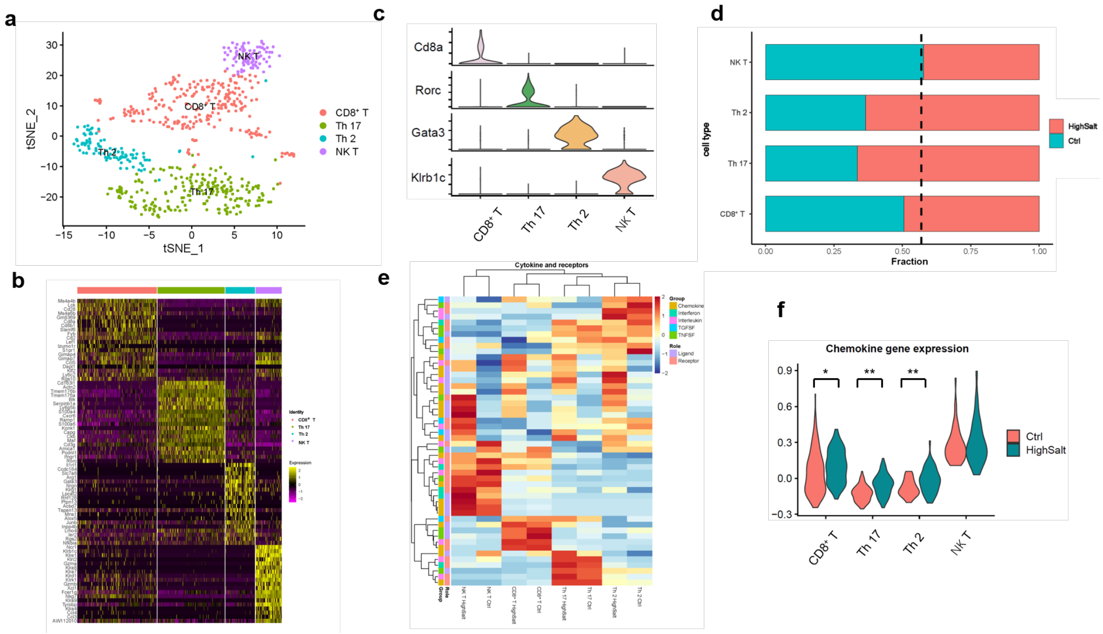

Supplemental Figure 9 Characteristics of aortic T cells and their changes in Hypertension. (a) t-SNE plots of the T cells subpopulations from control and hypertensive tissue (control, n = 447 cells. hypertension, n =587 cells. CD8$^+$T, n= 390 cells; Th17, n= 364 cells; Th2, n=159 cells; NK T, n=121 cells). (b) Heatmap showing the top 10 genes [by average Log(fold change)] for each T cells subpopulation. (c) Expression levels of T cells subpopulation marker genes. (d) Bar graph of the composition of each T cells subpopulation (dashed black line, expected proportion of cells in the control group). (e) Mean expression of cytokine and receptors genes in T cluster. (f) Chemokine gene set score of T cells subpopulations differentiates control and hypertensive. *Mann–Whitney U test P<0.05, **Mann–Whitney U test P<0.01.
